# Arsenic–silicon priming of rice (*Oryza sativa* L.) seeds influence mineral nutrient uptake and biochemical responses through modulation of Lsi-1, Lsi-2, Lsi-6 and nutrient transporter genes

**DOI:** 10.1038/s41598-018-28712-3

**Published:** 2018-07-09

**Authors:** Ehasanullah Khan, Meetu Gupta

**Affiliations:** Ecotoxicogenomics lab, Department of Biotechnology, Jamia Millia Islamia, New Delhi, 25 India

## Abstract

Silicon (Si) has attracted substantial attention because of its beneficial effect on plants during abiotic stress, including stress due to arsenic (As). We here report that priming rice seeds with As and Si together, helped the plant to sustain As stress for longer period. We examined Si induced tolerance against As in rice seedlings at short (7 d) and long (15 d) exposure periods under As^(III)^ and Si treatments since their germinating stage. Results showed that the expression of As^(III)^ transporter genes *OsLsi1*, *OsLsi2* and *OsLsi6 *was more in As^(III)^ + Si treatment as compared to control and Si treatment, but lower than As^(III)^ alone treatments. The gene expression was maximum in shoot and root at 15 d over 7 d under both As^(III)^ and As^(III)^ + Si treatment, which ultimately leads to decreased accumulation of As in the presence of Si. Morphological characters, antioxidant capacity, oxidative stress marker (MDA), stress modulators (cysteine, proline), and enzymes related with ascorbate-glutathione cycle significantly altered during As^(III)^ + Si treatment at both exposure periods. Further, macro and micronutrient contents also improved with Si, and differentially regulated 12 key genes (*NR*, *NiR*, *AMT*, *NR*, *GS*, *GOGAT*, *PT*, *PHT1*, *PHT2*, *APase*, *KAT1* and *HAK10)* related with NPK transport and utilization. Results highlight that Si priming of seeds along with As^(III)^ influences growth positively of As-stressed rice.

## Introduction

Arsenic (As) being a non-essential and non-threshold carcinogen, not only limits the growth of crop plants, but also contaminates the food chain. Rice (*Oryza sativa* L.) paddies contaminated by As is a staple crop over half the world population. Consumption of As contaminated rice results into many health ailments including arsenosis and cancer in several As contaminated regions of south Asian nations. Due to higher availability of As in anaerobic flooded soil, and also due to greater transport of As(III) across aqua-glyceroporins rice efficiently accumulates more As than other cereal crops^1^. Presence of As in soil/water reduces the nutrient content in rice plant resulting into impaired growth and development of crop^2^. The regulation of As content in rice plant is necessary to avoid toxicity, and plant roots being the primary site of accumulation plays important role in translocation and maintaining various cellular and metabolic processes. Inorganic As species largely exist in two forms in the environment, arsenate (As^V^) an oxidized form, and arsenite (As^III^) in a reduced form^3^. As^(V) ^is taken up by plant root from soil via phosphate transporter, whereas nodulin26-like intrinsic protein (NIP) is a major entry route for As^(III)^^4^. As interferes with various physiological, biochemical and molecular processes in plants and ultimately cause cell death^5,6^. As^(V)^ potentially replace Pi (inorganic phosphate) from the critical biochemical reaction of various metabolic (i.e., glycolysis, oxidative phosphorylation), biosynthetic process of cell (i.e., phospholipid metabolism), and cellular signalling (i.e., protein phosphorylation/dephosphorylation)^3^. As^(III)^ is a thiol reactive metalloid and act as a cross linking agent by binding up to three sulfhydryl group of antioxidants, like GSH and phytochelatein (PC). The stability of As^(III)^-thiol complex depends on the number of cysteine residue available in enzymes, protein and polypeptides^7^. It is well documented that As exposure in plants, leads to oxidative stress by generating reactive oxygen species (ROS), that can perturbed normal cellular process by damaging cell membrane, protein, enzyme and nucleic acid^8^. To combat oxidative stress, plant cells are well equipped with cellular antioxidant capacity, which includes enzymatic and non-enzymatic components^9^. Apart from various antioxidant enzymes, several stress markers and modulators such as cysteine, proline, malondialdehyde (MDA) and H_2_O_2_ contents indicate the level of injury and its protection^10,11^. However, response of all the detoxification machineries are influenced by As mobility, bioavailability, toxicity, presence of other ligands and nutrients^12^.

On the other hand, silicon (Si) is second most abundant element on earth. Studies revealed its beneficial role for plant growth and development, and protection from various biotic and abiotic stresses^13,14^. Among various gramineous plants, rice is able to accumulate Si higher than 10% of the shoot dry weight depending on soil Si concentration^14^. The significance of Si uptake, particularly in rice is due to specific uptake system of rice roots, which facilitates the transport of silicic acid across the plasma membrane. Si have been assessed to reduce As accumulation, improved antioxidant defence, and restrict photosynthetic impairment in rice^2,15^, however, few reports are available which highlights the significance of Si in growth recovery over short and long periods under As stress^15,16^. Low silicon rice 1*(Lsi1)* and low silicon rice 2 *(Lsi2)* are Si influx and efflux transporters, respectively, transports Si from root epidermis into the root steel, and then shoot via xylem sap. Both transporters belong to the family of aquaporin channel proteins, and constitutively express in rice roots and shoot. *Lsi1* and *Lsi2* are localised in distal and proximal side of both exodermis and endodermis cells of plasma membrane, respectively^17,18^. Furthermore, study on *Lsi1* and *Lsi2* transporter genes in rice under As^(III)^ treatment revealed that these transporters serve as major entry pathway for As^(III)^ transport in rice plant^19,20^. Uptake by roots through *Lsi 1* and *Lsi 2*, Si is translocated to the aboveground parts of the plant. *Lsi 6*, a homolog of *Lsi 1* involved in xylem unloading, and polarly localized in the adaxial side of the xylem parenchyma cells in the leaf sheath and leaf blades^21^. Si reduces the As^(III)^ accumulation in rice, and subsequently mitigates phytotoxicity, induced by As^(III)15^,and also counters the oxidative stress enhancing the antioxidant defence system^11^.

In addition to Si, plants require adequate amounts of macronutrients such as nitrogen (N), phosphorus (P), potassium (K), calcium (Ca), sulphur (S), magnesium (Mg); and micronutrients such as boron (B), chlorine (Cl), manganese (Mn), iron (Fe), zinc (Zn), copper (Cu), molybdenum (Mo), nickel (Ni) for their growth. The macro and micro nutrient plays crucial role in the biosynthesis and structure of nucleic acid, enzyme and proteins^22,23^. Deficiency of any nutrient affects the growth and development of plant. Arsenic is known to affect the plant adversely and alters the nutrient content, which are essential for human health. Rice, being a staple food considered as the principal source of nutrients, and As reduces the nutrient content in rice grains^24^. It also affect the expression of genes of N, P, and K metabolizing enzymes such as nitrate reductase (NR), nitrite reductase (NiR), glutamine synthetase (GS) and glutamate synthetase (GOGAT)] including their transporters such as NRT and AMT^25^. Although, the uptake, transport, and assimilation of N, P, and K in plants are well documented^26–28^, but the underlying mechanism in the presence of Si and As is still unclear. The availability of nutrients depends on the strategies for optimization of mineral nutrient transport through modulation of transporters activities. A co-operative system mediated by both efflux and influx Si transporters, along with other nutrient transporters required for the efficient mineral uptake. Recently, it has been reported that Si supplementation under As stressed in *Brassica* species enhances various macro and micro nutrient content^11^. Hence, the underlying mechanism through which the replenishment of macro and micro nutrients takes place in plants and particularly in rice plants is essential

Role of Si in mitigation of As toxicity has been reported^11,15^, however, to get an insight into the identification of plausible mechanisms responsible for providing differential stress response in rice plant, expression of different genes related with Si transportation and nutrients have been analysed under short (7 d) and long term (15 d, since germination) exposure of Si along with As. The presented work evaluates time dependent responses of Si priming since germination stage in the presence of As^(III)^ at both exposure period. We hypothesized that Si priming at germinating stage of seed along with As^(III)^, helped the plant to recover from As^(III)^ toxicity through modulation in gene expression of As/Si and nutrient transporters, which can alter the transportation of elements. The findings of the present study will address the following questions (i) Does Si priming at early level of seed germination provide tolerance against As^(III)^ toxicity (ii) Whether, Si + As^(III)^ combination reduces As accumulation and affects nutrient content through altering the gene expression pattern of different transporters

## Results

### Recovery of plant growth and physiological parameters under As(III) + Si combination

Growth parameters such as seed germination and shoot - root length was determined to measure the ameliorating effect of Si under As^(III)^ stress. Compared with control, As^(III)^ treatment decreased seed gemination by 42%, As^(III)^ + Si combination increased seed germination by 40%, compared with As^(III)^ treatment alone (Table 1). At 7 and 15 d after As^(III)^ treatment, shoot and root length decreased, however, addition of Si improved shoot and root length by 50% and 60% at 7 d, and 52% and 42%, respectively at 15 d over As^(III)^ alone treated plant. (Table 1; see Supplementary Fig. S2). Overall, phenotypic characteristics significantly ameliorated in the presence of Si as root length improved at 7 d, while at 15 d shoot growth enhanced.

**Table 1:** Effect of Si on percentage seed germination and shoot-root length in *Oryza sativa* var. IR64 treated with or without As^(III)^ at 7 and 15d exposure period. Different letters represent significant differences between each treatment at *p* < 0.05. The small latter (i.e. a, b, c and d) indicates the significant difference between treatment in same tissue type while capital latter (i.e. A and B) indicates the significant difference between duration among the same treatment in same tissue type.

Treatment	Seed germination (%)	Length (cm) 7 Day		Length (cm) 15 Day	
		Shoot	Root	Shoot	Root
C	95 ± 0.56^c^	8.0 ± 0.60^Ac^	6.0 ± 0.12^Ac^	15 ± 0.48^Bc^	13.8 ± 0.95^Bc^
Si	98 ± 0.44^c^	8.5 ± 0.65^Ac^	7.5 ± 0.15^Ad^	16 ± 0.41^Bd^	14.6 ± 0.65^Bc^
As	56 ± 0.61^a^	4.0 ± 0.31^Aa^	2.0 ± 0.13^Aa^	7.2 ± 0.25^Ba^	4.5 ± 0.18^Ba^
As + Si	78 ± 0.56^b^	6.0 ± 0.40^Ab^	3.2 ± 0.05^Ab^	11 ± 0.51^Bb^	6.4 ± 0.10^Bb^

**Figure 1:**
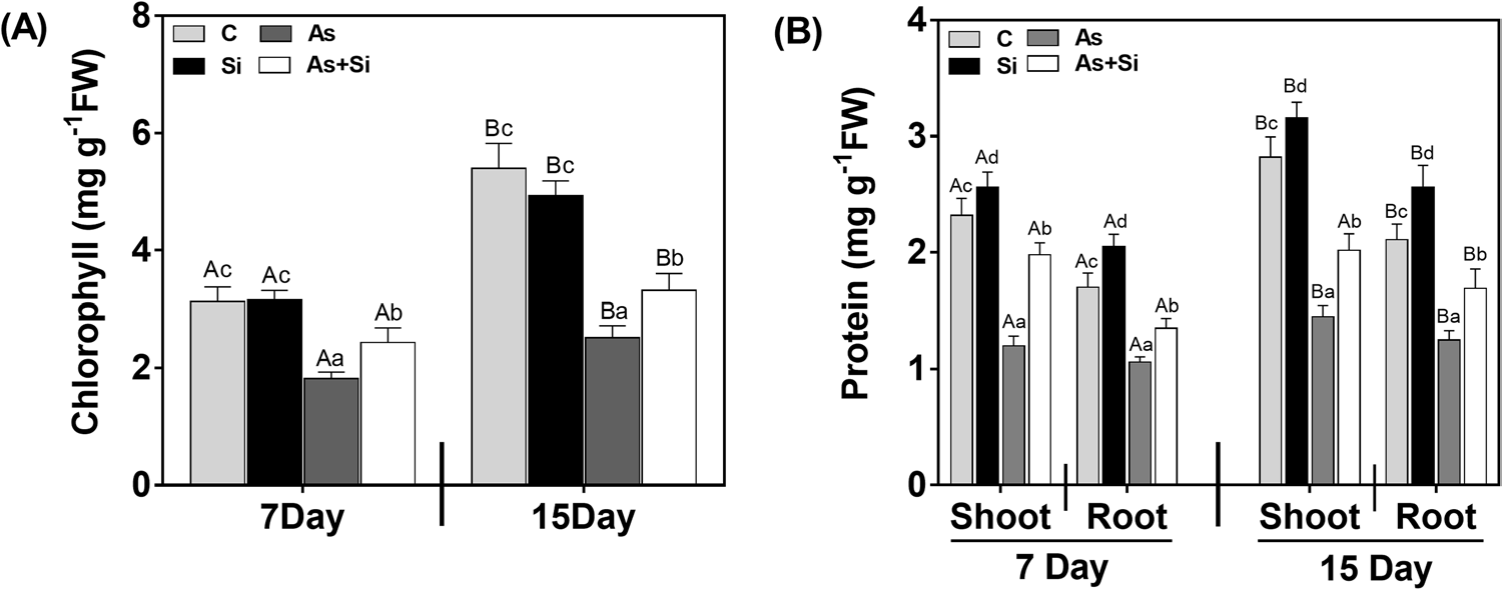
Effect of Si supplementation on (**A**) chlorophyll (**B**) protein content in shoot and roots of *Oryza sativa* var. IR64 at 7 and 15 d under As^(III)^ stress. All values are means of three replicates (n = 3, ±SD) and repeated twice. Different letters indicate significant mean difference (*p* < 0.05) between each treatment. The small latter (i.e. a, b, c and d) indicates the significant difference between treatment in same tissue type while capital latter (i.e. A and B) indicates the significant difference between duration among the same treatment in same tissue type.

Under As^(III)^ stress, chlorophyll and protein content decreased significantly at days 7 and 15 d compared with control (Fig. 1A,B). However, upon As^(III)^ + Si treatment total chlorophyll content significantly increased at both the exposure periods (31% at 7 d and 33% at 15 d), compared to As^(III)^ alone treatment. Protein content also increased significantly after application of As^(III)^ + Si by 65% and 27% in shoots and roots, respectively at 7 d. While, 39% (shoots) and 35% (roots) increase was observed at 15d, compared with As^(III)^alone treatment.

### Application of Si alters As accumulation and improves nutrient content in plants

As and Si content in their respective treatments remarkably increased both in roots and shoots at both the exposure periods (Table 2). In plants, treated with Si alone had higher Si content in shoots and roots at both exposure periods and As^(III)^ treatment (Table 2). Arsenic content in only As^(III)^ treated plants was more in roots (1.256 mg g^−1^DW) than shoots (0.825 mg g^−1^DW) at 15 d, while at 7 d no significant difference was observed between shoots (0.637 mg g^−1^DW) and roots (0.686 mg g^−1^DW) As accumulation. Application of Si with As^(III)^ leads to significant decrease in shoot by 66% and 49%, and in root it was 35% and 44% at 7 and 15 d, respectively, as compared to As^(III)^alone (Table 2). Overall, As content was higher in shoots and roots at 15 d compared to 7 d.

**Table 2:** As and Si accumulation in shoot and root of *Oryza sativa* var. IR64; four treatments were used (C-control, 150 µM As^(III)^, 5 mM Si, 150 µM As^(III)^ + 5 mM Si). The data represented as an average of three replicates and repeated twice. Different letters represent significant differences between each treatment at *p* < 0.05. The small latter (i.e. a, b, c and d) indicates the significant difference between treatment in same tissue type while capital latter (i.e. A and B) indicates the significant difference between duration among the same treatment in same tissue type.

	7 Day				
		C	Si	As	As + Si
Shoot	Si	1.01 ± 0.01^Aa^	20.71 ± 0.05^Ac^	—	12.52 ± 1.52^Ab^
	As	0.002 ± 0.001^Aa^	0.001 ± 0.0001^Aa^	0.637 ± 0.06^Ac^	0.216 ± 0.11^Ab^
Root	Si	0.54 ± 0.07^Aa^	13.43 ± 0.0^Ad^	1.88 ± 0.15^Ab^	10.54 ± 1.51^Ac^
	As	0.002 ± 0.0001^Aa^	0.001 ± 0.0001^Aa^	0.686 ± 0.027^Ac^	0.412 ± 0.07^Ab^
	**15 Day**				
Shoot	Si	1.23 ± 0.01^Aa^	24.31 ± 0.71^Bb^	2.10 ± 0.02^Bb^	16.35 ± 1.27^Bc^
	As	0.002 ± 0.0001^Aa^	0.001 ± 0.0001^Aa^	0.825 ± 0.01^Bc^	0.414 ± 0.02^Bb^
Root	Si	0.63 ± 0.02^Ba^	17.25 ± 0.54^Bd^	1.94 ± 0.03^Ab^	15.26 ± 0.02^Bc^
	As	0.003 ± 0.0001^Aa^	0.002 ± 0.0001^Aa^	1.256 ± 0.01^Bc^	0.724 ± 0.30^Bb^

Nitrogen (N), phosphorus (P) and potassium (K) are considered as primary macronutrient, necessary for growth and development of rice plant. Analysis in the present study revealed that N, P and K content after As^(III)^ treatment decreased at both exposure periods (7 and 15 d), in shoots and roots (Table 3), however, longer exposure (15 d) showed recovery in shoots P and K content (15% P and 2% K as compared to 7d). Application of As^(III)^ + Si, increased NPK content in both shoots and roots of rice at both exposure periods, as compared to As^(III)^alone (a recovery of 2% N, 16% P and 9% K in shoots, 7% N, 25% P and 4% K in roots was observed at 15 d, compared to 7 d). Additionally, four micronutrients (Mn, Fe, Cu, and Zn) were also decreased significantly (*P* < 0.05) in rice plant treated with As^(III)^alone, in shoots and roots at 7 and 15 d (Table 4). Overall, results indicated that macro-micronutrient content decreased in both shoot and root when plant were treated with As^(III) ^only. On the other hand, co-application of Si along with As^(III)^, could overcome the deficiency of nutrients caused due to toxicity of As^(III)^.

**Table 3:** Nitrogen (N), Phosphorus (P) and Potassium (K) content in shoot and root of *Oryza sativa* var. IR64 under As^(III)^ and Si supplementation at 7 and 15d exposure period. Values are mean (±S.D) of three independent replicates (n = 3). Different letters represent significant differences between each treatment at *p* < 0.05. The small latter (i.e. a, b, c and d) indicates the significant difference between treatment in same tissue type while capital latter (i.e. A and B) indicates the significant difference between duration among the same treatment in same tissue type.

Treatment	7 Day					
	Shoot			Root		
	N	P	K	N	P	K
C	7.68 ± 0.10^Ac^	4.27 ± 0.12^Ac^	10.3 ± 0.12^Ac^	6.65 ± 0.10^Ac^	3.25 ± 0.05^Ab^	9.82 ± 0.05^Ac^
Si	7.99 ± 0.23^Ac^	4.65 ± 0.20^Ac^	10.5 ± 0.56^Ac^	6.46 ± 0.01^Ac^	3.98 ± 0.04^Ab^	9.98 ± 0.02^Ac^
As	5.18 ± 0.1^Aa^	2.72 ± 0.13^Aa^	6.60 ± 0.14^Aa^	4.75 ± 0.22^Aa^	2.33 ± 0.10^Aa^	6.46 ± 0.07^Aa^
As + Si	6.26 ± 0.20^Ab^	3.32 ± 0.48^Ab^	8.83 ± 0.44^Ab^	5.05 ± 0.26^Ab^	2.52 ± 0.03^Aa^	7.83 ± 0.05^Ab^
	**15 Day**					
	**Shoot**			**Root**		
C	7.95 ± 0.05^Ac^	5.38 ± 0.21^Bb^	10.85 ± 0.54^Ac^	6.52 ± 0.06^Ac^	4.12 ± 0.42^Bc^	10.08 ± 0.12^Bc^
Si	7.95 ± 0.11^Ac^	6.08 ± 0.19^Bc^	11.82 ± 0.42^Bc^	6.71 ± 0.12^Ac^	4.81 ± 0.75^Bc^	10.16 ± 0.01^Bc^
As	5.16 ± 0.1^Aa^	3.14 ± 0.12^Ba^	6.78 ± 0.12^Aa^	4.48 ± 0.03^Aa^	2.55 ± 0.01^Aa^	6.39 ± 0.02^Ac^
As + Si	6.38 ± 0.15^Bb^	3.86 ± 0.15^Aa^	9.01 ± 0.15^Bb^	5.39 ± 0.01^Bb^	3.15 ± 0.01^Bb^	8.15 ± 0.02^Bb^

**Table 4:** Manganese (Mn), Iron (Fe) Copper (Cu) and Zink (Zn) content in shoot and root of *Oryza sativa* var. IR64 under As^(III)^ and Si supplementation at 7 and 15d exposure period. Values are mean (±S.D) of three independent replicates (n = 3). Different letters represent significant differences between each treatment at *p* < 0.05. The small latter (i.e. a, b, c and d) indicates the significant difference between treatment in same tissue type while capital latter (i.e. A and B) indicates the significant difference between duration among the same treatment in same tissue type.

	7Day							
	Shoot				Root			
	C	Si	As	As + Si	C	Si	As	As + Si
Mn	0.068 ± 0.004^Ac^	0.070 ± 0.003^Ac^	0.034 ± 0.001^Ba^	0.044 ± 0.003^Bb^	0.052 ± 0.002^Aa^	0.056 ± 0.014^Aa^	0.024 ± 0.013^Ab^	0.036 ± 0.001^Bc^
Fe	0.442 ± 0.024^Aa^	0.472 ± 0.072^Aa^	0.286 ± 0.020^Ab^	0.367 ± 0.020^Bc^	0.308 ± 0.007^Aa^	0.314 ± 0.052^Aa^	0.195 ± 0.046^Bb^	0.233 ± 0.026^Bc^
Cu	0.035 ± 0.003^Aab^	0.040 ± 0.006^Ab^	0.027 ± 0.002^Aa^	0.030 ± 0.001^Ba^	0.022 ± 0.002^Aa^	0.025 ± 0.002^Aa^	0.022 ± 0.002^Ba^	0.024 ± 0.012^Aa^
Zn	0.121 ± 0.027^Ac^	0.144 ± 0.045^Bd^	0.041 ± 0.010^Ba^	0.085 ± 0.001^Bb^	0.123 ± 0.016^Bc^	0.162 ± 0.041^Bd^	0.072 ± 0.001^Ba^	0.101 ± 0.002^Bb^
	**15 Day**							
	**Shoot**				**Root**			
	**C**	**Si**	**As**	**As + Si**	**C**	**Si**	**As**	**As + Si**
Mn	0.072 ± 0.001^Bc^	0.072 ± 0.002^Ac^	0.026 ± 0.001^Aa^	0.033 ± 0.002^Ab^	0.065 ± 0.012^Bb^	0.068 ± 0.001^Bb^	0.021 ± 0.001^Aa^	0.028 ± 0.001^Aa^
Fe	0.478 ± 0.051^Ab^	0.523 ± 0.040^Bb^	0.221 ± 0.025^Aa^	0.292 ± 0.050^Aa^	0.326 ± 0.007^Bb^	0.361 ± 0.043^Bb^	0.152 ± 0.056^Aa^	0.171 ± 0.062^Aa^
Cu	0.038 ± 0.002^Ab^	0.040 ± 0.001^Ab^	0.024 ± 0.005^Aa^	0.028 ± 0.002^Aa^	0.025 ± 0.003^Ab^	0.029 ± 0.001^Ab^	0.019 ± 0.001^Aa^	0.021 ± 0.001^Aa^
Zn	0.196 ± 0.011^Bd^	0.127 ± 0.036^Ac^	0.037 ± 0.002^Aa^	0.082 ± 0.001^Ab^	0.100 ± 0.04^Ac^	0.112 ± 0.037^Ac^	0.053 ± 0.002^Aa^	0.096 ± 0.001^Ab^

### Altered gene expression of silicon transporters (OsLsi1, OsLsi2 and OsLsi6) under As(III) and Si treatment

**Figure 2:**
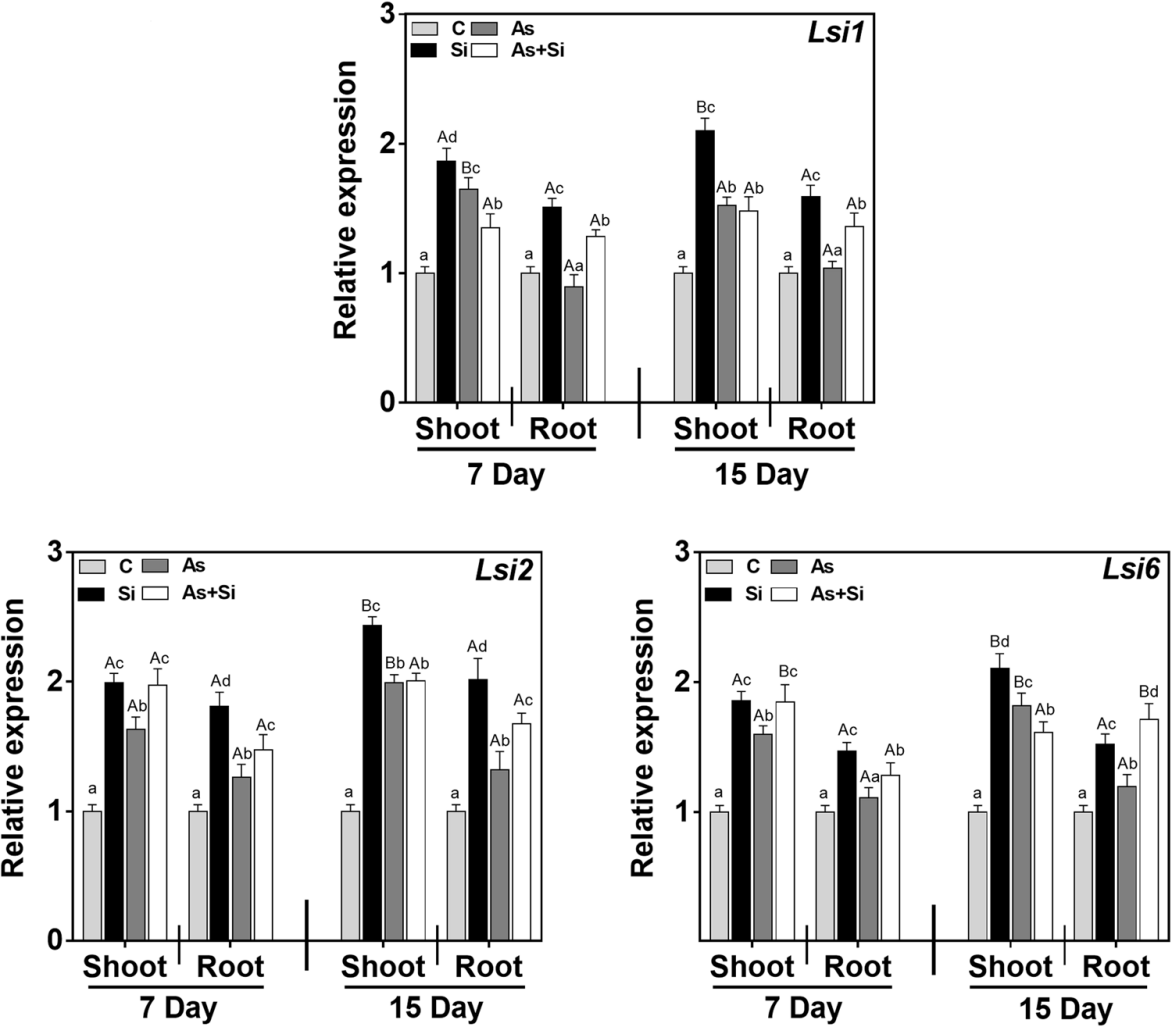
Effect of Si, As^(III)^, and As^(III)^ + Si on the relative expression of Si transporter (*Lsi1*, *Lsi2 and Lsi6*) in shoot and root of *Oryza sativa* var. IR64 at 7 and 15 d. All values are means of three replicates (n = 3, ±SD) and repeated twice. Different letters indicate the significant mean difference (*p* < 0.05) between each treatment. The small latter (i.e. a, b, c and d) indicates the significant difference between treatment in same tissue type while capital latter (i.e. A and B) indicates the significant difference between duration among the same treatment in same tissue type. The endogenous actin gene of rice was used to normalize the genes relative to control.

To gain an overview of the effect of different treatments, relative gene expression of *OsLsi1*, *OsLsi2 and OsLsi6* were examined by using qRT-PCR. The results showed up regulation of *OsLsi1*, *OsLsi2* and *OsLsi6* on As^(III)^ alone by 1.99, 1.63 and 1.97 fold in shoots, while 1.10, 0.99, and 1.28 in roots, respectively at 7 d (Fig. 2). Under As^(III)^ + Si treatment, the expression of above three genes was relatively higher than control but lower than As^(III)^ alone at 7 d in both shoots and roots (Fig. 2). Similarly, up regulation of *OsLsi1*, *OsLsi2* and *OsLsi6* was also observed by As^(III)^ treatment at 15 d by 2.23, 1.99 and 2.00 fold in shoots, while 1.81, 1.32, 1.67 fold in roots, respectively (Fig. 2). The expression of *OsLsi1*, *OsLsi2* and *OsLsi6* was more in shoots than roots. Furthermore, the expression of these genes was more in As^(III)^ + Si treatment as compared to control and Si alone but lower than As^(III)^ alone. Overall, the expression was maximum in shoots and roots at 15 d exposed plant than 7 d both in As^(III)^ and As^(III)^ + Si treatment.

### Silicon mediated differential gene expression of transporters and enzymes involved in nutrient (N, P, K) absorption and utilization

**Figure 3:**
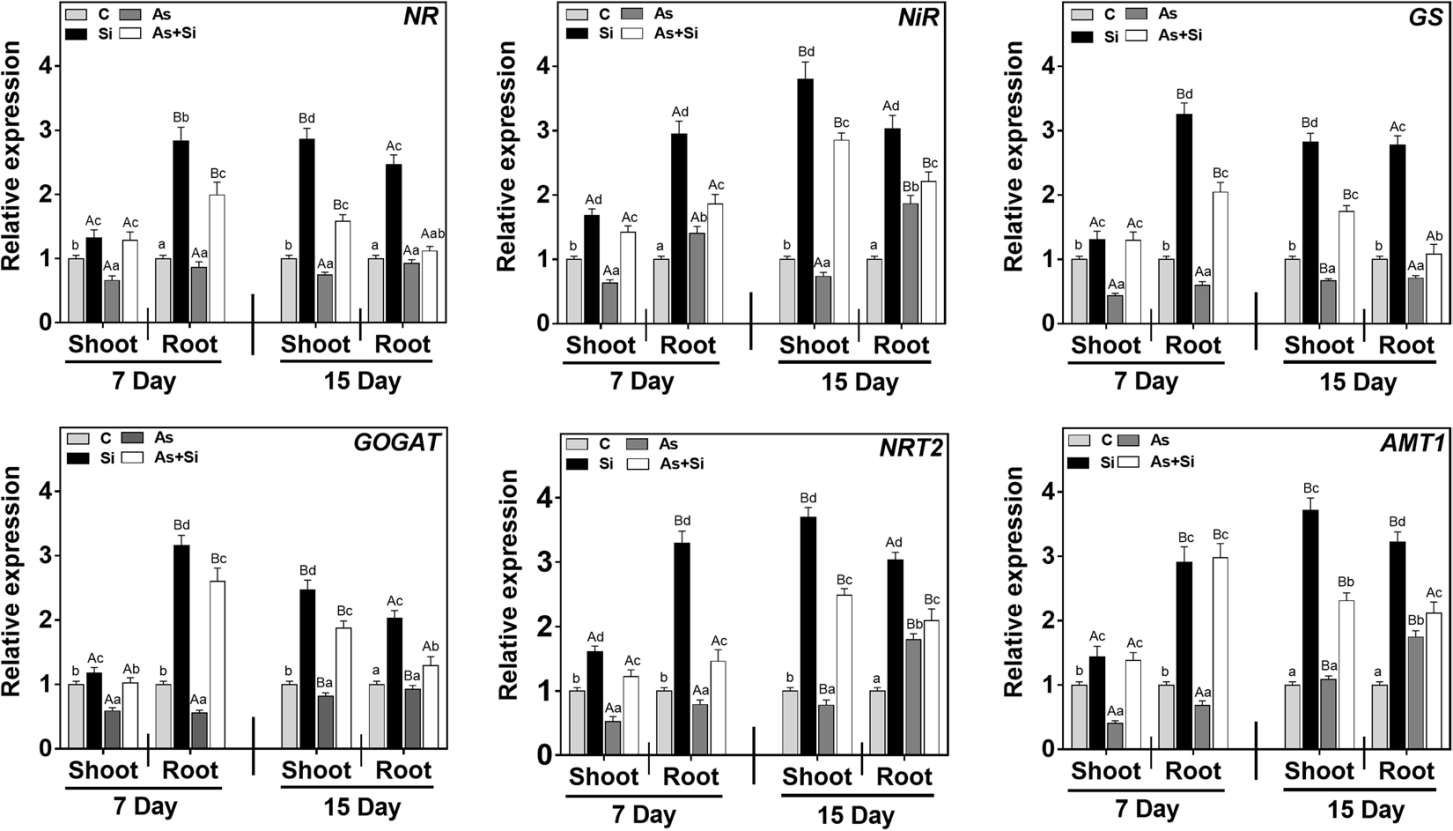
Differential expression of nitrogen absorption and assimilation related genes indicate their role under As and Si exposure in shoot and root of *Oryza sativa* var. IR64 at 7 and 15 d. Figure shows relative expression profile of six differentially regulated genes (nitrate reductase (*NR*), nitrite reductase (*NiR*), glutamine synthetase (*GS*), glutamate synthase (*GOGAT*), high affinity nitrate transporter protein (*NRT2*) and high affinity ammonium transporter protein (*AMT1*). All values are means of three replicates (n = 3, ±SD) and repeated twice. Different letters indicate the significant mean difference (*p* < 0.05) between each treatment. The small latter (i.e. a, b, c and d) indicates the significant difference between treatment in same tissue type while capital latter (i.e. A and B) indicates the significant difference between duration among the same treatment in same tissue type. The endogenous actin gene of rice was used to normalize the genes relative to control.

**Figure 4:**
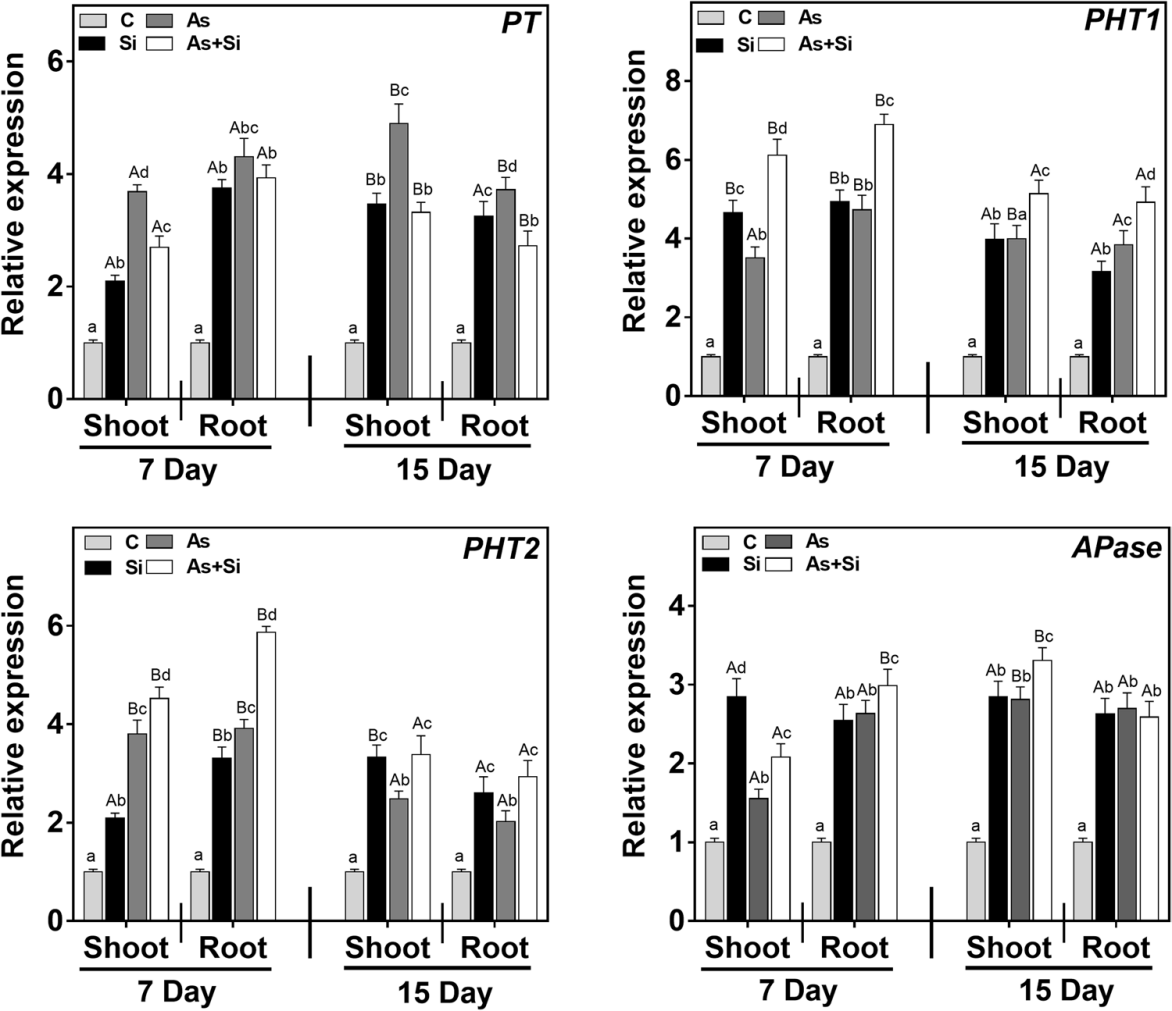
Differential expression of phosphorus absorption and assimilation related genes indicate their role under As and Si exposure in shoot and root of *Oryza sativa* var. IR64 at 7 and 15 d. Figure shows relative expression profile of phosphate transporter (*PT*), high-affinity phosphate transporter 1 (*PHT1*), high affinity phosphate transporter 2 (*PHT2*) and acid phosphatases (*APase*). All values are means of three replicates (n = 3, ±SD) and repeated twice. Different letters indicate the significant mean difference (*p* < 0.05) between each treatment. The small latter (i.e. a, b, c and d) indicates the significant difference between treatment in same tissue type while capital latter (i.e. A and B) indicates the significant difference between duration among the same treatment in same tissue type. The endogenous actin gene of rice was used to normalize the genes relative to control.

**Figure 5:**
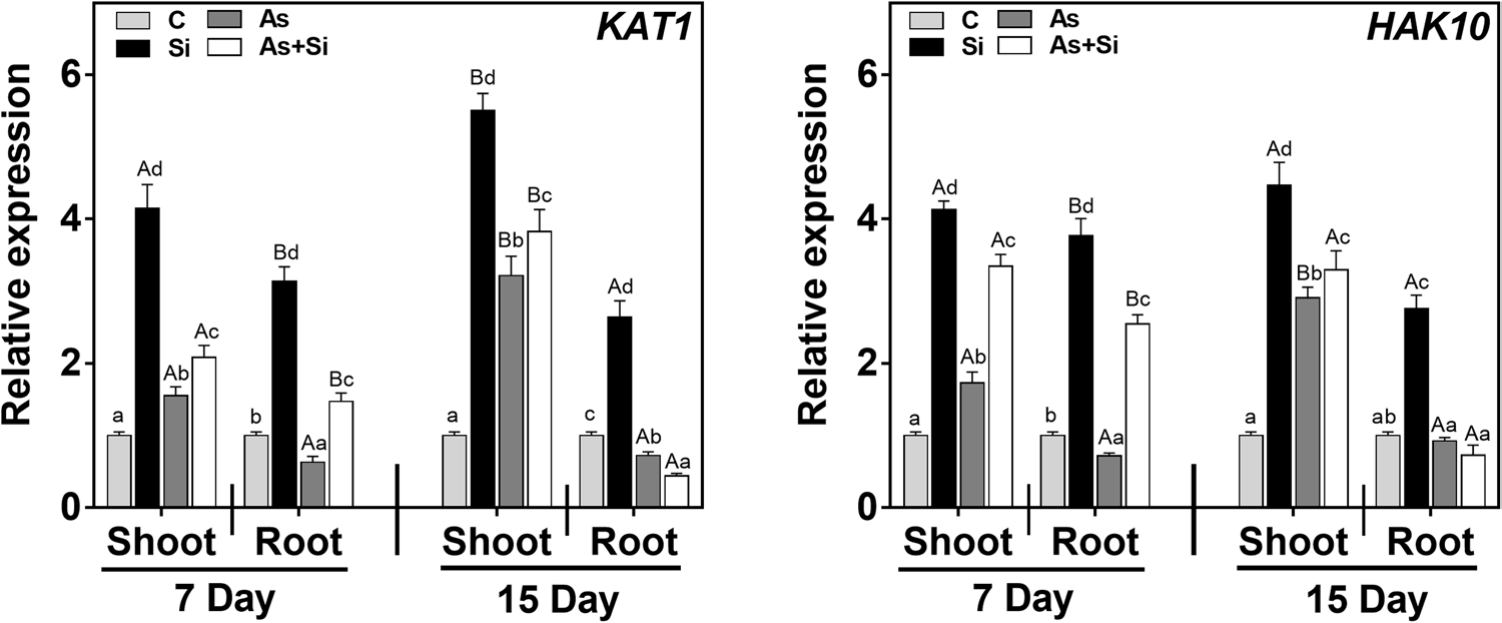
Differential expression of potassium absorption and assimilation related genes indicate their role under As and Si exposure in shoot and root of *Oryza sativa* var. IR64 at 7 and 15 d. Figure shows relative expression profile of potassium channel protein (*KAT1*) and potassium transporter protein (*HAK10*). All values are means of three replicates (n = 3, ±SD) and repeated twice. Different letters indicate the significant mean difference (*p* < 0.05) between each treatment. The small latter (i.e. a, b, c and d) indicates the significant difference between treatment in same tissue type while capital latter (i.e. A and B) indicates the significant difference between duration among the same treatment in same tissue type. The endogenous actin gene of rice was used to normalize the genes relative to control.

Priming seeds with Si along with As^(III)^ modulates the expression of nutrient related genes. qRT-PCR of 12 genes in rice shoots and roots (7 and 15 d) was carried out to determine the relative mRNA expression of macronutrients, 6 Nitrogen (N), 4 Phosphorus (P) and 2 Potassium (K) transporters and utilization genes. The relative mRNA expression of genes *NR*, *GS* and *GOGAT* was down-regulated in shoots and roots at days 7 and 15 in As^(III)^alone, being maximum in roots, compared to all the treatments (Fig. 3). In As^(III)^ treated plants, expression of *NiR*, *NRT2* and *AMT1* in roots at 15 d, were observed to be up-regulated over control, while in shoots the expression of these genes were down regulated at both exposure periods (Fig. 3). However, upon As^(III)^ + Si treatment, relative mRNA expression level of all six genes (*NR*, *NiR*, *GS*, *GOGAT*, *NRT2* and *AMT1*) were up regulated both in shoots and roots at 7 and 15 d over As^(III)^ alone and control (Fig. 3). Phosphorus transporters genes (*PT*, *PHT1*, *PHT2*, and *APase*) were up regulated in As^(III) ^alone treated plants at both exposure periods (Fig. 4). In As^(III)^ + Si combination, expression of *PT* down regulated relative to As^(III)^ alone both in shoots and roots at 7 and 15 d (Fig. 4). However, other genes of P transporter were found to be up regulated under As^(III)^ + Si combination, compared to control and As^(III)^ alone, both in shoots and roots at 7 and 15 d. Potassium (K) transporter genes (*KAT1* and *HAK10*) up regulated in shoots at all the treatments at days 7 and 15 (Fig. 5). The expression of these genes was higher in Si treated plants, over control/ As^(III)^/and As^(III)^ + Si, The relative mRNA level of *KAT1*and *HAK10* genes down-regulated in roots at 7 d and 15 d (Fig. 5), under As^(III) ^stress as compared to other treatments.

### Si treatment decreased As(III)-induced LOX activity, MDA content and alters antioxidant activities

**Figure 6:**
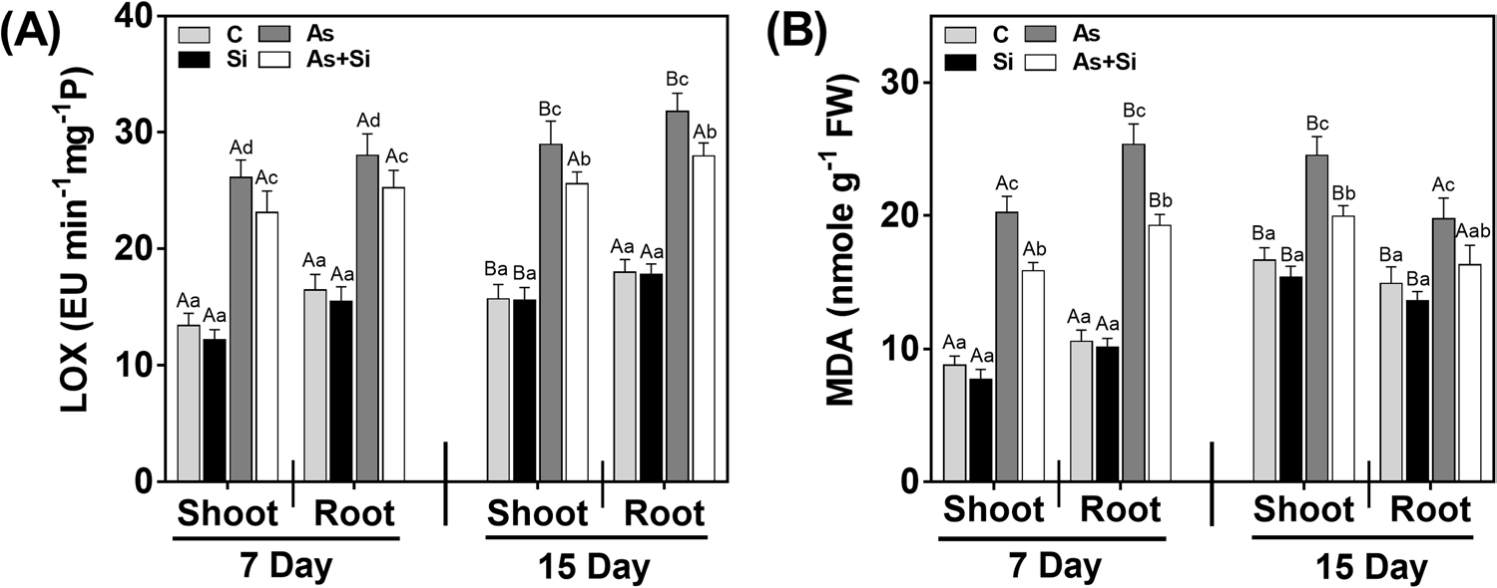
Effect of Si and As^(III)^ alone and in combination on (**A**) LOX enzyme activity (**B**) MDA content in shoot and roots of *Oryza sativa* var. IR64 at 7 and 15 d. All values are means of three replicates (n = 3, ±SD) and repeated twice. Different letters indicate the significant mean difference (*p* < 0.05) between each treatment. The small latter (i.e. a, b, c and d) indicates the significant difference between treatment in same tissue type while capital latter (i.e. A and B) indicates the significant difference between duration among the same treatment in same tissue type.

LOX, an oxidative enzyme leads to the formation of MDA. In As^(III)^ treated plant, LOX activity increased both in shoots and roots at 7 and 15 d (Fig. 6A). Significant reduction was observed under As^(III)^ + Si combination in both shoots and roots as compared to As^(III)^ alone treated plant at 7 and 15 d. However, LOX activity was observed with non-significant difference between Si alone and control for both exposure periods. As^(III) ^stressed rice plants showed enhancement in MDA level by 130% and 139% at 7 d, while 47% and 32% at 15 d for shoots and roots, respectively (Fig. 6B). However, increase in MDA content was more at 7 d in both shoots and roots than 15 d. This higher increase in MDA level of As^(III)^-stressed plants decreased under As^(III)^ + Si combination by 21% and 24% at 7 d (shoots), and 18% and 17% at 15 d (roots) over As^(III) ^alone.

**Figure 7:**
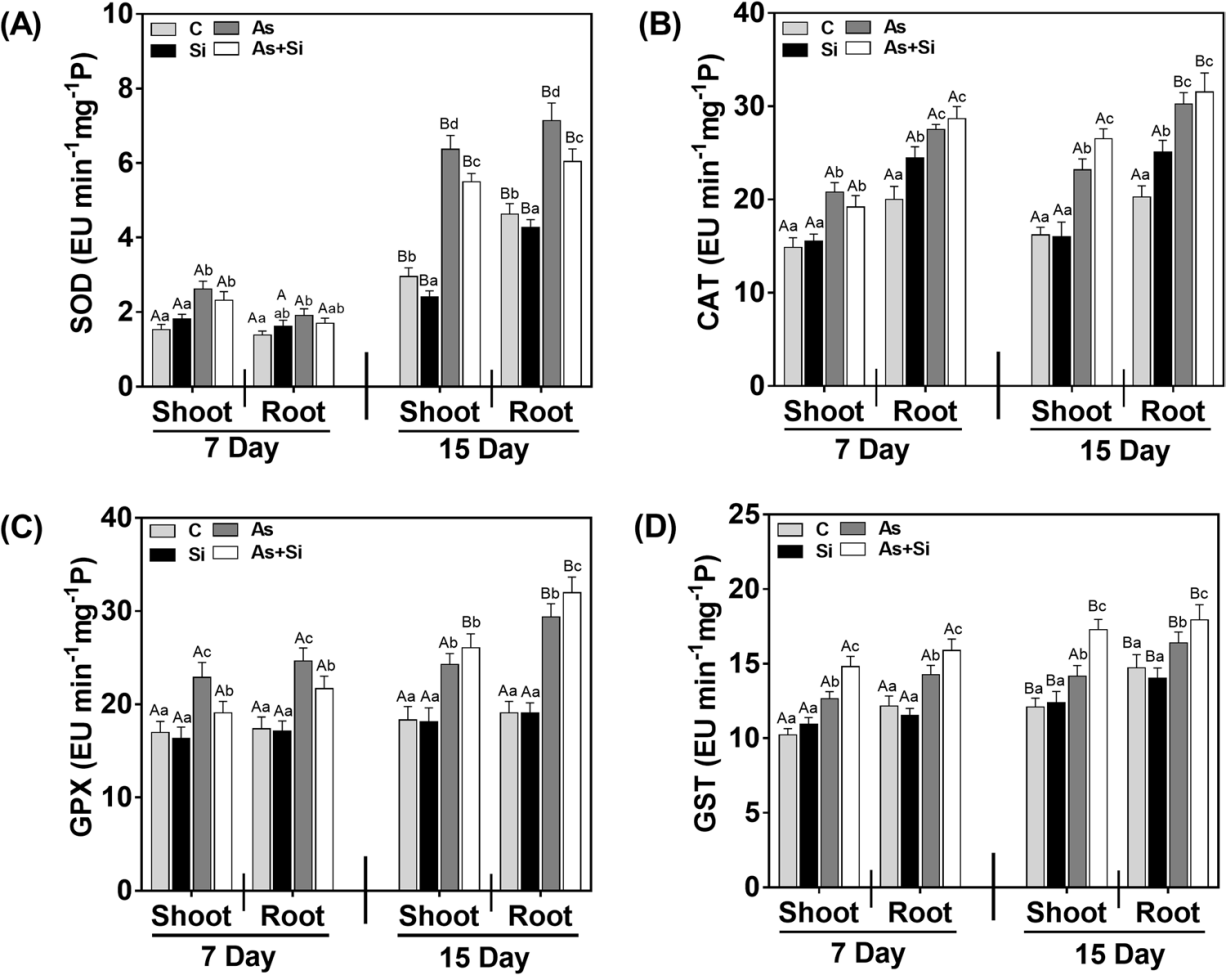
Effect of Si, As^(III)^, and As^(III)^ + Si on (**A**) SOD (**B**) CAT (**C**) GPX and (**D**) GST activity in shoot and roots of *Oryza sativa* var. IR64 at 7 and 15 d. All values are means of three replicates (n = 3, ±SD) and repeated twice. Different letters indicate the significant mean difference (*p* < 0.05) between each treatment. The small latter (i.e. a, b, c and d) indicates the significant difference between treatment in same tissue type while capital latter (i.e. A and B) indicates the significant difference between duration among the same treatment in same tissue type.

The antioxidant system of plant helps to counter up oxidative stress generated by ROS. In the present study, increased SOD activity observed in As^(III)^ + Si treated plants in shoots and roots at both exposure periods. However, activity decreased in shoots roots at days 7 and 15, compared to As^(III) ^alone treated plants (Fig. 7A). The activity of CAT (a heme-containing enzyme), which detoxifies H_2_O_2_ to H_2_O and O_2_, increased at 7 d by 39% and 52%and43% and 44% at 15 din shoots and roots, respectively under As^(III)^ stress, being more in roots (Fig. 7B). As^(III)^ + Si treatment increased CAT activity at both exposure periods in shoots and roots, however, increase was insignificant except in shoots (14%) at 15 d compared to As^(III) ^alone (Fig. 7B).

GPX activity in As^(III)^ stressed plants showed increase by 34% and 41% in shoots and roots, respectively at 7 d, whereas at 15 d it was 32% in shoots and 53% in roots (Fig. 7C). On the other hand, As^(III)^ + Si lowered the GPX activity by 16% in shoots and 12% in roots as compared to As^(III) ^alone treated plants at 7 d (Fig. 7C). However, at 15 d no significant decrease was observed in As^(III)^ + Si treated plants as compared to As^(III)^ alone treatment. The result of GST activity upon As^(III)^ treatment, increased significantly (*p* < 0.05) at 7 d (23% in shoots and 17% in roots), and 15 d (17% in shoots and 11% in roots) (Fig. 7D). Further increase in GST activity was also observed in plants treated with As^(III)^ + Si. The increase was 44% in shoot and 30% in root at 7 d, while at 15 d it was 53% and 97% in shoot and root, respectively (Fig. 7D). On the other hand, As^(III)^ + Si combination also increased GST activity over the As^(III)^ treated plants (16% in shoot and 11% in root at 7 d, and 21% and 9% in shoot and root, respectively at 15d) (Fig. 7D).

### Ascorbate-glutathione cycle enzymes APX, MDHAR, GR and stress modulators, cysteine and proline

**Figure 8:**
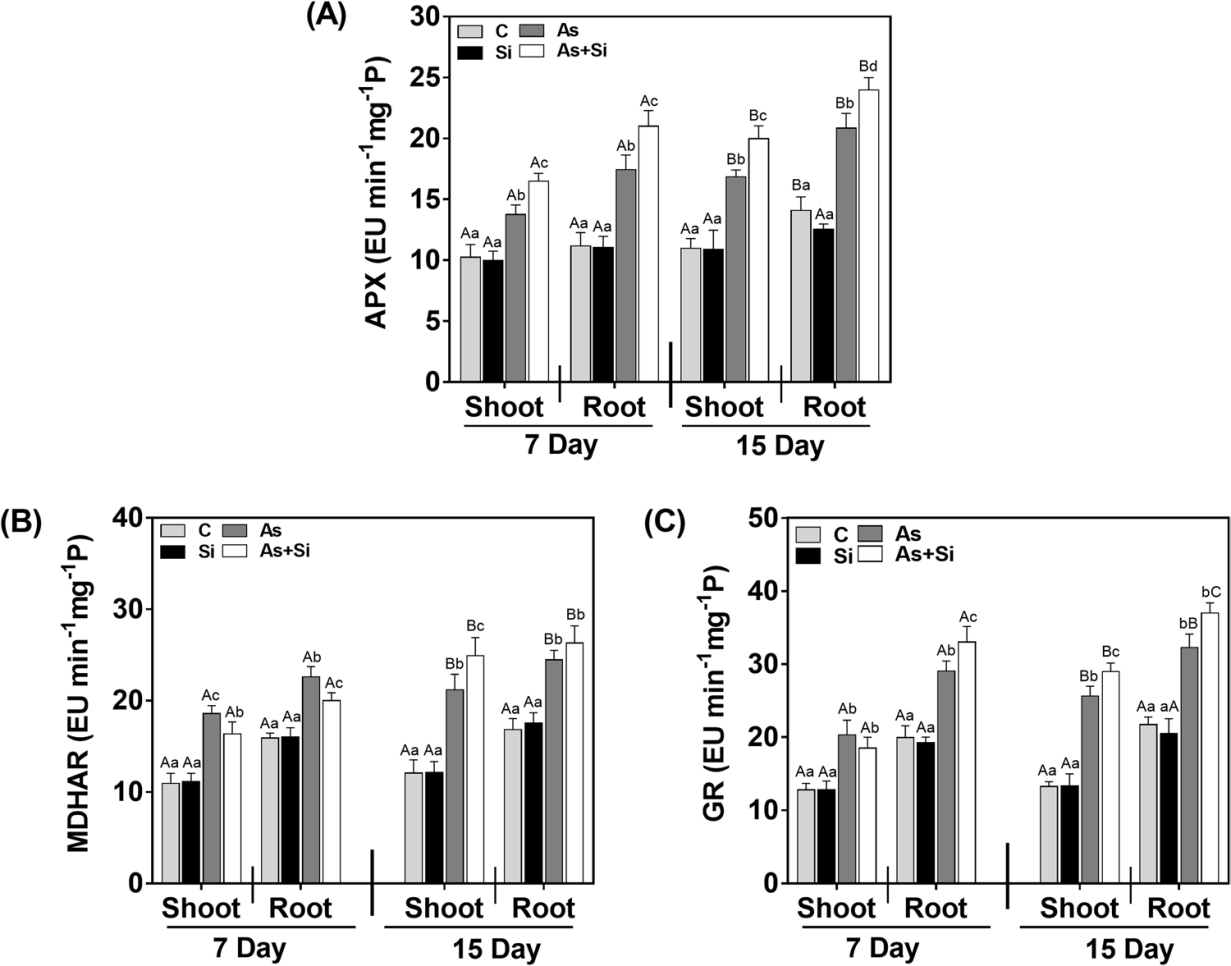
Effect of Si supplementation on (**A**) APX (**B**) MDHAR, and (**C**) GR activity in shoot and roots of *Oryza sativa* var. IR64 at 7 and 15 d under As^(III)^ stress. All values are means of three replicates (n = 3, ±SD) and repeated twice. Different letters indicate the significant mean difference (*p* < 0.05) between each treatment. The small latter (i.e. a, b, c and d) indicates the significant difference between treatment in same tissue type while capital latter (i.e. A and B) indicates the significant difference between duration among the same treatment in same tissue type.

APX, the first enzyme of ascorbate-glutathione cycle that scavenge H_2_O_2_ in cell increased in As^(III) ^alone treated plants roots at 7 d, whereas decreased at 15 d (Fig. 8A). APX activity in plants treated with As^(III)^ + Si, increased at 7 d (51% in shoots and 87% in roots) and 15 d (72% in shoots and 70% in roots) compared with As^(III) ^alone treated plants. MDHAR is an enzyme which reduces MDHA (monodehydroascorbate) to AsA (ascorbic acid) that serves as an electron donor to reduce H_2_O_2_ to H_2_O and O_2_ with the help of APX. In As^(III)^ treated plants, activity of MDHAR increased at days 7 and 15 (Fig. 8B). On the other hand, under As^(III)^ + Si combination, further increase in MDHAR activity was observed at 7 and 15 d (Fig. 8B). Similarly, increase in GR activity was also observed in As^(III)^ + Si treated shoots and roots at both exposure periods (Fig. 8C). However, greater GR activity observed in As^(III)^ + Si treated plants, compared with control at 15d (118% in shoots and 70% in roots) than 7 d, (44% in shoots and 65% in roots)

**Figure 9:**
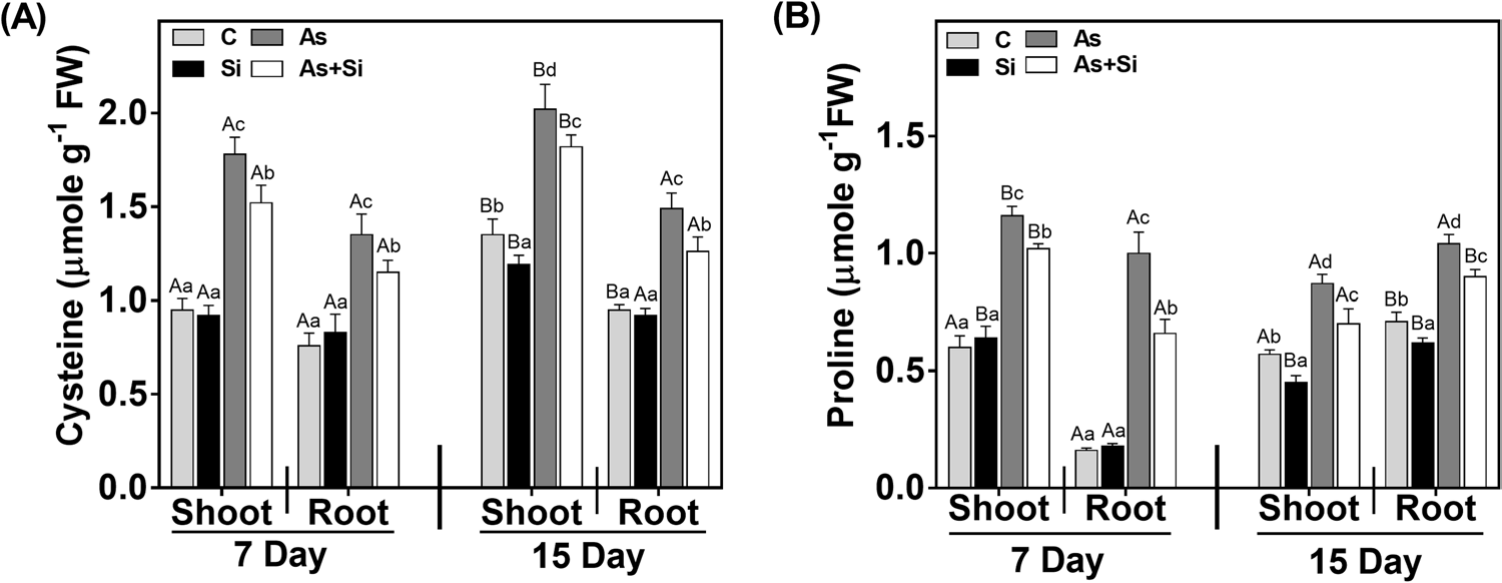
Effect of Si, As^(III)^, and As^(III)^ + Si on (**A**) cysteine (**B**) proline content in shoot and roots of *Oryza sativa* var. IR64 at 7 and 15 d. All values are means of three replicates (n = 3, ±SD) and repeated twice. Different letters indicate the significant mean difference (*p* < 0.05) between each treatment. The small latter (i.e. a, b, c and d) indicates the significant difference between treatment in same tissue type while capital latter (i.e. A and B) indicates the significant difference between duration among the same treatment in same tissue type.

Cysteine and proline content were measured as stress modulators. Both cysteine and proline content significantly (*P* < 0.05) increased in As^(III)^ treated plants for both shoots and roots at days 7 and 15 (Fig. 9A,B). As^(III)^ + Si combination showed further decrease in cysteine content by 17% and 18% at 7 d in shoots and roots, respectively, however, at 15 d increase was observed 10% in shoots and 18% in roots over As^(III)^alone being more in root (Fig. 9A). However, when, proline content in As^(III)^ + Si compared with As^(III)^ alone, a significant decrease was observed at 7 d and increase at 15 d (Fig. 9B).

Fold change of all biochemical parameters are summarized in Supplementary Table (S2).

## Discussion

Arsenic is known to inhibit overall growth and development of plant by affecting tissue proteins, enzymes, and other cellular functions^29^. It has been reported that Si exposure helps in the growth and development of plants under biotic and abiotic stress, including metals such as As^30–34^. Plant cells have various transporters which facilitates the uptake and accumulation of As, and other mineral nutrients inside the plant *viz*. aquaglyceroporins, ATP binding (ABC) cassette transporter, nitrogen and phosphate transporters^26^. It is important to reduce the accumulation of unwanted elements through regulation of these transporters, as they play prominent role in intracellular metal homeostasis. Rice being an efficient silicon accumulator among all crop plants, and the same Si transporters are shared by As too, the current study gives new insight into how through modulation in gene expression of As/Si and nutrient transporters, Si provide protection to rice seedlings against As^(III)^ toxicity. Si- dependent amelioration of As toxicity since germination stage of seeds has not been analyzed in detail. The presented findings highlight the significance of Si induced recovery of stressed plant as early response towards As^(III)^ stress resulted in a decrease in the overall growth of plant at both exposure periods. However, Si + As^(III)^ combination enhanced the growth positively. Improved growth of plant signifies the ameliorating action of Si during As^(III)^ toxicity. Previous studies also showed that Si exert beneficial effects on plant growth and development by alleviating the biotic and abiotic stresses such as salt, drought, disease and heavy metals^11,35,36^. In the present study, decrease was observed for both chlorophyll and protein content under As^(III)^ stress, which is directly correlated with the endogenous As concentration^10,37^, while Si supplementation along with As^(III)^ significantly increased both the contents. Similar study has been reported in *Brassica* and rice, which is in concordance with our results. Further, visible phenotypic condition of plant was better at 7 d, but increase in chlorophyll and protein content was observed at longer duration (15d). Probably, this might be due to Si mediated lowering of As^(III)^ toxicity by decreasing its internal content in plant or by providing the rigidity to plant tissue and improved photosynthesis^2,38^. Further, increase in protein content in As^(III)^ + Si treatment might be associated with the binding of Si with amino acids to form specific proteins or its engagement in the formation of DNA and functioning of mRNA^39,40^.

Presented results revealed toxicity in rice plant was related with the amount of As/Si over the exposure periods, and its effect on the overall growth of plant. Treatment with Si reduced As uptake and enhanced uptake of Si in rice seedlings^15^. Interestingly, it has been reported that As shares the Si transport pathway to enter the plant via *OsLsi1* and *OsLsi2*, and their expressions are inversely proportional to external Si concentration^17,18,41^. Our results showed Si mediated decrease in As content both at short (7 d) and long exposure periods (15 d), being more in roots compared to shoots. These results are comparable with the previous reports, where presence of Si reduced the uptake of metals in rice^15,42,43^. The possible mechanisms for reduced uptake of As can be explained that Si strongly binds to cell wall components, induced structural alterations which leads to the blockage of the apoplasmic transport, and restricts the entry of metal^36,44^. This pattern also signifies the alleviation of As^(III)^ toxicity (as roots were not damaged) through lowering MDA content and other stress related parameters (antioxidant enzymes), as compared to As^(III)^ alone treated plant. Further, lower As content in As^(III)^ + Si treated plant than As^(III)^, might be due to Si mediated down-regulation of *OsLsi1*and *OsLsi2* in As^(III)^ + Si treated plant as compared to As^(III)^ alone. Similarly, decrease in As content relative to the expression of *OsLsi1* and *OsLsi2* has been reported in rice under Si supplementation along with As^2^.

Earlier studies reported that Si and As transport is mediated by Si transporters Os*Lsi1* and Os*Lsi2* in rice. The *OsLsi1*, an influx Si transporter localized on distal side of exodermis while *OsLsi2* and *OsLsi6* are localized on proximal side of endodermis of rice root and function as efflux Si transporter. In rice root, the *OsLsi* gene expressed constitutively but their expression varies greatly in the presence and absence of Si and As in the growth medium^17,41,45^. In the present study, up regulation of Os*Lsi1*, Os*Lsi2* and Os*Lsi6* in shoot and root at both 7 and 15 d was positively correlated with As content in As^(III)^ stressed plants, suggesting the role of *OsLsi* (*Oryza sativa* low silicon rice) transporters in As transport in rice. Furthermore, in As^(III)^ + Si treated plant up regulation of these genes over As^(III)^ treatment alone did not correlate with the As content in the plant, indicating that the level of *OsLsi* gene expression might not be sufficient to accumulate As in the presence of Si, and this might be due to the higher affinity of these genes (Os*Lsi1*, *OsLsi2 and OsLsi6*) towards Si than As. Under both Si and As^(III)^ + Si treatment the accumulation of Si was maximum than As, while down regulation of *OsLsi1*, *OsLsi2 and OsLSi6* gene was observed in their respective treatments at both exposure periods in shoots and roots as compared to As^(III) ^treatment/ control. In another study, similar pattern of *Lsi* gene expression and relative Si accumulation was observed in rice that supports our result^2^.

**Figure 10:**
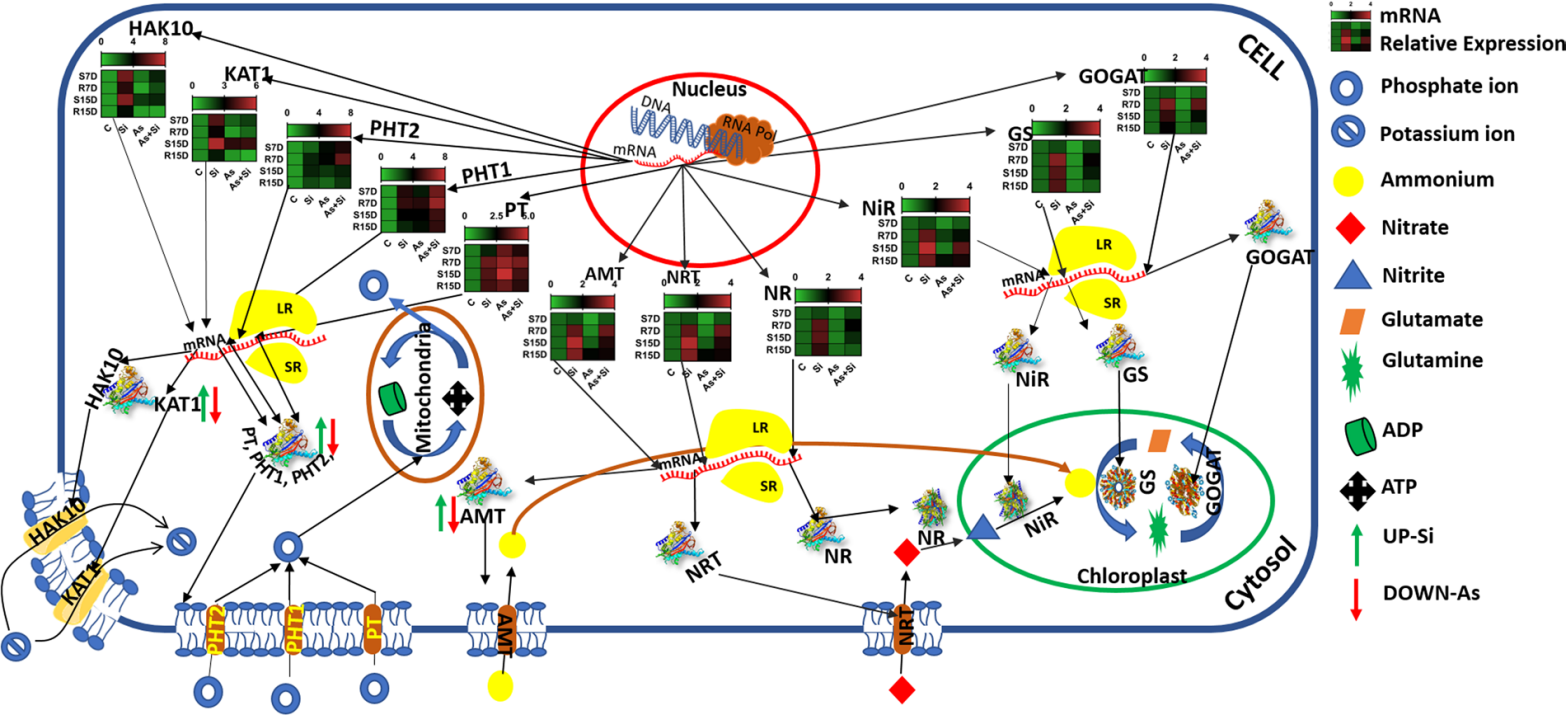
A schematic presentation of key genes involved in nitrogen, phosphorus and potassium (NPK) transport and their utilization system in a plant cell. Heat map represents the relative mRNA expression under different treatments.NO_3_^-^are transported through *NRT* and NH_4_^+^ through *AMT* in the cytosol. NO_3_^−^ is reduced to NO_2_^−^ by enzyme *NR* in the cytosol while NO_2_^−^ is transported into the chloroplast and reduced to ammonium ion (NH_4_^+^) by enzyme *NiR*. The NH_4_^+^ ion takes part in glutamine synthesis and glutamine converts into glutamate by enzymes *GS* and *GOGAT* in the chloroplast. The inorganic phosphate (P*i*) uptake was carried out by *PT*, *PHT1* and *PHT2* that leads to reversible synthesis of ATP and ADP in mitochondria. Potassium ions are taken up by *KAT1* and *HAK10*. The arrows, UP (green) and DOWN (red) in the figure is showing the expected possible mechanism where silicon (Si) and arsenic (As) might be responsible for up and/or down expression and modification of proteins that affects the function(s) of enzymes and transporters. (NO_3_^−^ Nitrate; NRT-High Affinity Nitrate Transporter; AMT-High Affinity Ammonium Transporter; NR-Nitrate Reductase; NiR-Nitrite Reductase; GS-Glutamine Synthetase; GOGAT-Glutamate Synthetase; PT-Phosphate Transporter; PHT1 and PHT2- High Affinity Phosphate Transporter 1 and 2; KAT1-Potassium Transport Protein; HAK10-Potassium Channel Protein).

Plant primary macro nutrients, nitrogen (N), phosphorus (P) and potassium (K) content have effective role in the plant growth and development. In this study, twelve genes responsible for NPK absorption and utilization were investigated. In plants, nitrate and ammonium is primary source of nitrogen. Plants regulate nitrate and ammonium intake by high affinity nitrate transporter (*NRT2*) and high affinity ammonium transporter (*AMT1-2*). Nitrate further reduced into nitrite by the enzyme nitrate reductase (NR) in cytosol and translocated to chloroplast, where it reduced into ammonium by another enzyme called nitrite reductase (NiR). The ammonium reduced from nitrite subsequently assimilated into glutamine and then glutamate by glutamine synthetase (GS) and glutamate synthetase (GAGOT). Abiotic stress such as salt, drought, extreme cold and heavy metal leads to the down regulation of *NRT2* and affects N uptake and assimilation in mustard and rice^25,46^. In the present study, result revealed downregulation of *NRT2* gene in roots at 7 d, while upregulation was observed in roots at 15 d during As^(III)^ stress, compared to control. However, the expression was not positively correlated with N content in roots that remains lower than control at 15 d. It has been reported that *NRT2* requires partner protein *NAR2* to facilitate nitrate transport^47^, and change/alteration in *NAR2* expression might be the reason for low N content in roots at 15 d. While in As^(III)^ + Si treated plant, upregulation was observed in *NRT2* gene, which was positively correlated with N content as compared to As^(III)^alone. It has been reported that Si enhance the N content in tomato and olea plant under salt stress^48,49^, which supports our result of Si mediated upregulation of *NRT2*, and increased N content. Plants accumulate ammonium more rapidly than nitrate when provided in equal concentration. In this study, we observed down regulation of *AMT1* in shoots and roots at 7 d under As^(III)^ stress. The down regulation of *AMT1* was also reported in rice, lotus and mustard during As, drought and cold stress^25,46,50^. Further, at 15 d we observed upregulation of *AMT1* in root under As^(III)^ stress, again the enhanced expression of *AMT1* during As^(III)^ stress was not positively correlated with N content in plant root. It has been reported that over expression of *AMT1* may causes poor growth and poor N content^27^. Furthermore, in As^(III)^ + Si and Si treated plant the expression of *AMT1* was higher than control/As^(III)^ alone along with growth and N content. Expression of *AMT1* was also regulated by ammonium concentration present in the solution, lower concentration leads to up regulation of *AMT1*^51^. The upregulation of *AMT1* might be due to the presence of lower ammonium concentration. A schematic presentation of key genes involved in nitrogen, phosphorus and potassium transport and their utilization system in a plant cell are presented in Fig. 10.

Once nitrate enter the plant it is converted into nitrite by NR. Our results revealed down regulation of *NR* and *NiR* in shoot and root at 7 and 15 d during As^(III)^ stress. Downregulation of *NR* was also observed in shoot and root of rice and brassica under As and salt stress^25,46^. During As^(III)^ + Si treatment enhanced expression of *NR* and *NiR* was observed as compared to control, but less N content in the same treatment. The reason might be associated with improper regulation of NR at transcriptional and translational level^52^ due to As^(III)^. In addition, downregulation of GS and GOGAT in both shoots and roots was observed during As^(III)^treatment, similar results are reported by Wang *et al*. (2010) in rice. Further, GS and GOAGT was upregulated in plants treated with As^(III)^ + Si, and this might be due to Si mediated reduction of As content and its toxicity. Pi uptake by diffusion is very slow process, therefore, plant requires high affinity energy driven phosphate transporter, and this role is facilitated by proteins that belong to *PHT* family protein^53,54^. It has been known, that internal Pi concentration influence the expression of *PHT* gene for accumulation of Pi from outer medium, and modification at post transcription and post translation level has also been proposed for *PHT*^55,56^. Presented data showed upregulation in *PT*, *PHT1*, *PHT2*and *APase* under As^(III)^ treatment, but less P content, and this might be due to failure of post transcription as well as post translation modification. It may also be speculated that the level of expression was not enough to overcome the As induced toxicity. However, during As^(III)^ + Si treatment the expression of *PHT* genes and P content becomes higher than As^(III)^ alone which might be associated with Si mediated reduction in cytotoxicity induced by As^(III)^ as Si has been known for alleviation of biotic and abiotic stress^14^. K transporter genes *KAT1* and *HAK10* expression, and K accumulation in plants treated with As^(III)^ showed similar pattern as P transporter genes. Similar reasons could be possible for low K uptake, while higher expression of gene controlling the accumulation of K in roots and shoots was observed. As^(III)^ + Si effects was contrary to As^(III)^alone and increased mRNA levels of K transporter as well as K content. The presented results suggest the role of Si to mitigate the As^(III)^ toxicity by lowering As content, and subsequently enhance the expression of NPK genes. It has been reported that Si plays a vital role by regulating various genes involve in the major physiological process during biotic and abiotic stress^57^. Overall, the expression of NPK transporter genes under As^(III)^ stress were maximum in roots than shoots at both exposure periods, being more for 15 d exposure period. However, NPK content was not positively correlated at some points with the expression of their relative genes. This decrease in NPK content might be associated with higher As content in roots than shoots that may lead to inhibition of folding of protein by binding with their thiol group, and alter the function of transporter protein^58,59^.

In plants, cysteine serve as a central metabolite that donate their sulphur to biosynthesis of various molecules such as amino acids, vitamins, Fe-S cluster, GSH and thiol-containing proteins^60^. An increase in cysteine content in plant under metal stress has been reported extensively in plants including rice^10,37,61^. Increased level of cysteine observed under As^(III)^ and As^(III)^ + Si treated plants, however, it was less under As^(III)^ + Si as compared to As^(III)^ alone plant, except at 15 d. The increase in cysteine content is closely associated with biosynthesis of sulphur containing compounds, and is also a part of phytochelatins, which plays role in heavy metal detoxification^10,15^. Previous work reported increased cysteine content under As^(III)^ + Si in rice and *Brassica* plant^11,15^. Contrary to the previous reports, less cysteine content in As^(III)^ + Si during short duration (7 d), indicates that it does not require this detoxification machinery due to protective role of Si, as Si supplemented with As^(III)^ since their germinating stage. However, longer duration (15 d) needs protection from As^(III)^ through the stimulation of glutathione and phytochelatins. Proline, an amino acid serves as an osmo-protectant under abiotic stress such as drought, salinity and heavy metal^10,62,63^. Less proline content was observed under As^(III)^ + Si as compared to As^(III)^alone which suggests that addition of Si from the germinating stage protected cells by keeping the proline content to an optimum level, and probably employed other osmo-protectants for As mitigation. In plants, heavy metal toxicity persuades the production of ROS, leading to the oxidative damage^9^. To combat metal incited oxidative damage, plants have evolved enzymatic and non-enzymatic anti-oxidative defence mechanisms^64^. The enzymatic antioxidant enzymes constitute SOD, CAT, GPX and ascorbate- peroxidase cycle enzyme such as APX, MDHAR and GR. SOD is known as the first antioxidant enzyme to detoxify highly toxic O_2_˙ˉ to less toxic H_2_O_2_ that further converts into H_2_O and O_2_ through CAT. Increased activity of SOD and CAT under As^(III)^ stress in roots and shoots were as observed at both exposure periods (7d and 15d). however, As^(III)^ + Si treatment decreased SOD and CAT activity as compared to As^(III)^ alone, except in shoots at 15 d, might be due to higher level of H_2_O_2_. Similar pattern was observed in rice and mustard plants under As and As + Si treatment^11,15^, respectively. Another important mechanism associated with the detoxification of heavy metal induced oxidative stress includes GPX and GST^65^. GPX and GST are known as stress enzymes that help to reduce toxic oxygen intermediate. The activity of GPX and GST enhanced under abiotic stress including heavy metals^66^. Current results showed marked increase in GPX and GST activities under As^(III)^ stress, being more in roots. This might be due to GSH-dependent peroxide scavenging, which helps in the reduction of oxidative damage, and membrane protecting role of Si through GST, preventing further damage of membrane. This is also in accordance with decreased MDA content under As^(III)^ stress. The result contradicts the observation by Tripathi *et al*.^15^, which showed decrease in GPX activity under As stress in rice. However, there are other reports in rice under Cu stress^65^ that supports our present observations. Furthermore, increased activities of GST and GPX under As^(III)^ + Si was also observed, which suggests that Si is preventing the oxidative damage by reducing the GSSG to GSH. This phenomenon however, was not confirmed by our experiment except some increase in GR activity that regulates GSH. In addition, 15 d treatments of As^(III)^ + Si enhanced most of the enzymes activities than 7 d. This might be due to increased nutrient content, that are not only required for biosynthesis of biomolecules, but also serve as co-factor for many enzymes.

The ascorbate-glutathione cycle enzyme involves APX, MDHAR and GR for maintaining the redox balance of AsA and GSH levels in plants under metal stress, and metabolise H_2_O_2_^9^. Activity of these enzymes (APX, MDHAR and GR) increased under As^(III)^ alone both in roots and shoots at 7 and 15 d. Similar results were observed in rice under cobalt^65^ and As treatment in rice^37^. Further, increase in APX, GR, and MDHAR activity under As^(III)^ + Si combination was observed over As^(III)^ treated plants. This suggests Si mediated increased role of these enzymes in regulating the ROS content. Moreover, decreased MDA content under Si supplementation under As^(III)^ stress, may be closely associated with cellular ROS level. Similar effect of Si was observed in rice under Zn and Cu stress^67,68^. Overall, data suggested that Si plays protective role in decreasing the ROS by enhancing the plants antioxidant capacity, and scavenging of As induced ROS, which might be correlated with ascorbate- glutathione cycle enzymes but not SOD and CAT. Accumulation of As demonstrated increased LOX activity and level of MDA content, indicating membrane damage in rice seedlings by peroxidation of membrane lipids. However, treatment with Si reduced both MDA content and LOX activity, and the reason might be due to lower ROS production and modulation of antioxidants mechanism involved in the protection of plant from As stress.

In conclusion, the findings of the present study revealed the differential impact of both exposure periods in the modulation of Si and NPK transporter genes in As^(III)^ stressed rice seedlings, which is ameliorated by Si priming along with As. Priming with Si and As^(III)^ together from germinating stage has been shown to be beneficial in alleviating As^(III)^ toxicity for both exposure periods, through the restriction of As accumulation, production of antioxidant and ascorbate-glutathione cycle related enzymes. Overall, seed priming with As^(III)^/Si together helped the plant to sustain for a longer period, however, phenotypic difference was not significant at both exposure periods. The Si induced alleviation of As stress from their germinating stage will help in the regulation As uptake via biological interactions, such as expression of various transporter genes and enzyme activities. This study gives new insight for identifying more rice genotypes, their transcriptome metabolite profiling and Si assisted plant responses.

## Methods

### Plant material and growth conditions

Rice seeds (*Oryza sativa* L. var. IR-64) obtained from Indian Agriculture Research Institute (IARI), Pusa, New Delhi, were surface sterilized in 1% (v/v) NaOCl for 1 min followed by washing with distilled water (DW). Preliminary screening to select better As^(III)^/Si combination during germination was done for different days (1–7) in the presence of low (50 µM), moderate (150 µM) and high (300 µM) As^(III)^ concentrations (see Supplementary Fig. 1). After selection of final concentration (150 µM As^(III)^), equal number of seeds (30) were treated with As^(III)^/ Si in triplicates (30 seeds in each Petri plate), and allowed for germination on a moist cotton bed in dark containing 5% Hoagland nutrient solution. After 2 Days, the germinated seeds were placed in trays having fixed PVC cups under hydroponic condition. The trays were transferred to light (a 16 h photoperiod) with a day/night temperature of 25 ± 2 °C in a controlled growth chamber with 70% relative humidity (RH),and further grown for 7 and 15-day (d)exposure periods. The treatment conditions included: (1) Control- containing only 5% Hoagland solution (2) Si(5 mM) as silicic acid (3) As^(III)^(150 µM) as NaAsO_2,_ and (4) As^(III)^(150 µM) + Si(5 mM). All the treatments were carried out in 3 replicates and All nutrient solutions were replaced twice in a week. After harvesting, each plant was washed thoroughly with DW, and stored in −80 °C or dried as required for experiments. While choosing As^(III)^ concentration, we opted for the concentration (150 µM) which significantly hampers plant growth and is based on our previous study^37^. The seeds were primed with As^(III)^ and Si since their germination due to two reasons:(i) in the field condition plants remains exposed to metal till the harvesting (ii) Si priming along with As improved the metabolic processes of seeds during germination under As stress (Supplementary Fig. S1). To mimic the field condition, we have chosen slightly higher concentration of As^(III)^ under hydroponic condition for long and short exposure periods.

### Morphological and physiological analysis

After 7 d, germinated seeds were scored and computed as follows: (No. of germinated seeds/number of total seeds) × 100. Root and shoot lengths (cm) were measured for both 7 and 15 d old plants using a metric scale to evaluate fold change in the various parameters, seeds were considered germinated when both the plumule and radicle were extended from their junction^65^. Plant height was determined by measuring the length from the bottom of the main stem to the end of the emerging third leaf. Chlorophyll content was analyzed following Arnon^69^. Leaves (100 mg) were homogenized in ice-cold acetone (80% v/v), centrifuged at 10,000 g for 10 min, absorbance of the supernatant was recorded at 663 and 645 nm. For protein estimation, fresh leaves and roots (0.2 g) were crushed and mixed with 2 ml phosphate buffer (pH 7.0), centrifuged at 10,000 g for 10 min at 4 °C. Protein content was measured following Bradford^70^ using bovine serum albumin (BSA) as a standard.

### Elemental analysis (As, Si, and nutrient contents)

To estimate As, Si and nutrient contents, harvested plants were rinsed with DW, roots and shoots separated, weighed, dried in oven at 60 °C until a constant dry mass was obtained. Equal amount of dried samples (ca. 200 mg) were acid digested, with 2 ml of each HNO_3_ and H_2_O_2_ on a hot plate till the samples converted into a fine residue. Total As, content was measured using an Inductively Coupled Plasma Mass Spectrometer (ICP-MS, Agilent 7500 USA) following standard protocol mentioned by Dubey *et al*.^71^. Used reference material for As was rice flour NIST with known spiked samples. The detection limit of As was 1 µg l^−1^. Silicon content was measured following colorimetric molybdenum blue method as described by Pandey *et al*.^11^. Total nitrogen content was estimated by employing the method of Yusuf *et al*.^72^. The plant material was digested with concentrated H_2_SO_4_, followed by its neutralization with NaOH and sodium silicate solutions. Nessler’s reagent was added, and absorbance was recorded at 525 nm on a spectrophotometer.

### Isolation of RNA, cDNA preparation and gene expression analysis

Total RNA was isolated shoots and roots of rice plants (7 and 15 d old) using Trizol reagent (Sigma Aldrich Life Science). The RNA was treated with DNaseI (Fermentas) and quantified by Nanodrop-spectrophotometer (Thermo scientific), and the quality was assessed using 1.2% agarose gel. Approximately, 2 µg of total RNA was used for first strand cDNA synthesis using RevertAid H Minus First Strand cDNA synthesis kit (Fermentas), as per manufacturer’s protocol. The qRT-PCR was performed in 384 well plate with SYBR Green using ABI Prism 7000 sequence detection system (Applied Biosystems, CA) as described previously Pandey *et al*.^73^. Samples from each treatment analysed in triplicate. The relative gene expression was analysed by using the 2^(ΔΔCt)^ method described by Livak *et al*.^74^, and presented as the fold change in gene expression or percentage of expression. The endogenous actin gene of rice was used as internal control. The primers used in qRT-PCR study are listed in Supplementary Table (S1).

### Biochemical analysis

Fresh samples of shoots and roots (0.2 g each) was homogenised in extraction buffer containing 50 mM phosphate buffer (pH 7.0), 100 mM KCl, 1 mM AsA, 5 mM β-mercaptoethanol, and 1% (w/v) TritonX-100 in pre-chilled pestle and mortar. The homogenate was centrifuged at 12,000 g for 15 min at 4 °C, and supernatants were collected to analyse enzyme activities.

SOD (EC 1.15.1.1) activity was measured according to Dhindsa *et al*.^75^ which is based on the inhibition of photochemical reduction of nitro blue tetrazolium (NBT). Formation of superoxide was noted at 560 nm and enzyme activity of SOD was measured as EU min^−1^mg^−1^ protein. CAT (EC 1.11.1.6) activity was measured according to Aebi^76^ with some modifications. Decrease in absorbance was recorded after every 1 min for up to 3 min at 240 nm. The CAT activity was expressed as EU min^−1^mg^−1^ protein. The activity of APX (EC 1.11.1.11) was determined according to Nakano and Asada^77^ by observing the decrease in absorbance at 290 nm for 1 min after adding H_2_O_2_ in a final volume of 1 ml reaction mixture. The activity was calculated by using extinction coefficient of 2.8 mM^−1^ cm^−1^.MDHAR (EC 1.6.5.4) activity was measured in a reaction mixture as described by Hossain *et al*.^78^. The oxidation rate of NADPH was recorded using 1U of AO (Ascorbate Oxidase) at 340 nm. LOX (EC 1.13.11.12) activity was measured according to Doderer *et al*.^79^. The increase in absorbance was recorded at 234 nm by using linoleic acid as substrate. GR (EC 1.6.4.2) activity was determined as described by Foyer and Halliwell^80^. The change in absorbance of NADPH was recorded at 340 nm. GST (EC 2.5.1.18) activity was measured according to Hossain *et al*.^81^. The increase in absorbance was recorded for 1 min at 340 nm after adding 1 mM 1-chloro-2,4-dinitrobenzen (CDNB) in a final volume of 1 ml reaction mixture. GPX (EC: 1.11.1.9) activity was measured^82^ by using H_2_O_2_ as a substrate. The change in absorbance at 340 nm for oxidation of NADPH was recorded as described by Elia *et al*.^82^.

Method of Gaitonde^83^ was used for cysteine estimation. Shoots and roots (0.5 g) were ground to a fine powder in 5 ml of chilled perchloric acid (HClO_4_) followed by centrifugation at10,000 g for 20 min as described by Pandey *et al*.^11^. Cysteine content was calculated from standard curve using L-cysteine (Himedia).Proline content was measured according to Bates *et al*.^84^. Plant tissue (0.5 g) were homogenized in 3% sulfosalicylic acid followed by centrifugation at 4,000 g for 10 min. Proline content was measured as described by Pandey and Gupta^85^. The amount of free proline concentration was determined from a standard curve. MDA content was estimated following Heath and Packer^86^. Plant tissue (0.2 g) were crushed and extracted in 2 ml with 1% concentration of TCA and centrifuged at 12,000 *g* for 10 min. The supernatant, was heated with TBA and the absorbance was recorded at two different wavelengths of 532 and 600 nm.

### Statistical analysis

Data were subjected to one-way analysis of variance (ANOVA). The resulted data are mean ± SD of three replicates (n = 3) of each treatment. Different letter indicates the significant difference between treatments at *p* ≤ 0.05 according to Duncan’s multiple range test. The small letters (a, b, c and d) indicates the significant difference between treatments, while capital letters (A and B) indicates the significant difference between durations between same treatment. All values present in the result section presented in % age are against their control values or otherwise.

## Electronic supplementary material

Supplementary Information
