# Supplementary material for: Arsenic–silicon priming of rice (Oryza sativa L.) seeds influence mineral nutrient uptake and biochemical responses through modulation of Lsi-1, Lsi-2, Lsi-6 and nutrient transporter genes
Source: Sci Rep. 2018 Jul 9;8:10301. doi: 10.1038/s41598-018-28712-3 (PMC6037781; doi:10.1038/s41598-018-28712-3)

**Arsenic–silicon priming of rice (*Oryza sativa* L.) seeds influence mineral nutrient uptake and biochemical responses through modulation of Lsi-1, Lsi-2, Lsi-6 and nutrient transporter genes**

Ehasanullah Khan and Meetu Gupta*

Ecotoxicogenomics Lab, Department of Biotechnology, Jamia Millia Islamia, New Delhi-25, India.

*Corresponding author

**Dr Meetu Gupta**,

Ecotoxicogenomics Lab,

Department of Biotechnology,

Jamia Millia Islamia, Jamia Nagar

New Delhi –110025

India

Tel +91 1126735142; 09818911750

*Email*: [meetu_gpt@yahoo.com](mailto:meetu_gpt@yahoo.com), [mgupta@jmi.ac.in](mailto:mgupta@jmi.ac.in)

**Table S1**. List of primer used for qRT-PCR analysis.

| **Key Genes** | **Source** | **Primer Sequence 5’-3’ direction** |
| --- | --- | --- |
| ***Lsi1*** | *Oryza sativa* | F-CGGTGGATGTGATCGGAACCA  R-CGTCGAACTTGTTGCTCGCCA |
| ***Lsi2*** | *Oryza sativa* | F-GAGTTCGACAACGTCTAATCGC  R-AGTACACGGTACATGTATACACG |
| ***Lsi6*** | *Oryza sativa* | F-AGGGAGCAGCAGCAAGA  R-AGGGAGTAGAGGGCGAAGGT |
| ***NR*** | *Oryza sativa* | F-GGAGAAGCCCACCAAG  R-CCCCATGAGATTCCAGAT |
| ***NiR*** | *Oryza sativa* | F-GAGATTGGAGGCACATAA  R -AGTCGAGCACTTCAGCATT |
| ***GS*** | *Oryza sativa* | F-GCGGTTGGCTATTGTT  R-ACCTGTTTTGCTGGAGTA |
| ***GOGAT*** | *Oryza sativa* | F-TCACGAATAAAAGAAAGGC  R-GACACGTCCATGCTCATC |
| ***NRT-2*** | *Oryza sativa* | F-GCACGGTGGCGATGAAA  R-GGAGATGGTGGTGAAGGAC |
| ***AMT-1*** | *Oryza sativa* | F-GTGGTGCTCGTCCATTTCC  R-CGTATTGCTGGTCTCGGTC |
| ***PT*** | *Oryza sativa* | F-ACAACTCCCTCGGCAACA’  R-CGTCGTCCAGCCTCTTCT |
| ***PHT-1*** | *Oryza sativa* | F-TGGTTCCTCCTTGACATCG  R-GCCCATCGCCAGCAT |
| ***PHT-2*** | *Oryza sativa* | F-CAGCAAGGTCGGGTGGAT  R-GAAGAAGGTGAGTGCGTAGAGC |
| ***APase*** | *Oryza sativa* | F-AAAGCACCCAGTGGAATC  R-ACCCTCGGAAAGTAAACG |
| ***KAT1*** | *Oryza sativa* | F-CACCTCCTTCATCATCCTGC  R-GCCCCACATCCTTTCTTTT |
| ***HAK10*** | *Oryza sativa* | F-AGGGAGCAGCAGCAAGA  R-AGGGAGTAGAGGGCGAAGGT |
| ***Actin*** | *Oryza sativa* | F-GACTCTGGTGATGGTGTCAGC  R- GGCTGGAAGAGGACCTCAGG |

**Table S2.** Comparison of **(A)** oxidative stress markers and stress modulator (MDA, Protein, Cysteine, Proline,). Enzymatic cellular antioxidants **(B)** SOD, CAT and GPX, GST, and **(C)** APX, MDHAR, and GR in shoot and roots of rice ver. IR-64 at 7 and 15 d in presence of As, Si and As+Si. All treatments were compared to control (C) seedlings. the negative **(-)** value indicate down fold.

**(A)**

| **Fold Change 7DAY** | | | | | | | | |
| --- | --- | --- | --- | --- | --- | --- | --- | --- |
|  | **Protein** | | **MDA** | | **Cysteine** | | **Proline** | |
|  | **Shoot** | **Root** | **Shoot** | **Root** | **Shoot** | **Root** | **Shoot** | **Root** |
| **C** | 0 | 0 | 0 | 0 | 0 | 0 | 0 | 0 |
| **Si** | 0.103 | 0.206 | -0.12132 | 0.240854 | -0.032 | 0.092 | 0.233 | 0125 |
| **As** | -0.483 | -0.376 | 1.305147 | 1.396341 | 0.600 | 0.513 | 0.066 | 5.251 |
| **As+Si** | -0.147 | -0.206 | 0.805147 | 0.820122 | 0.874 | 0.776 | 0.700 | 3.125 |
| **Fold Change 15DAY** | | | | | | | | |
|  | **Protein** | | **MDA** | | **Cysteine** | | **Proline** | |
|  | **Shoot** | **Root** | **Shoot** | **Root** | **Shoot** | **Root** | **Shoot** | **Root** |
| **C** | 0 | 0 | 0 | 0 | 0 | 0 | 0 | 0 |
| **Si** | 0.121 | 0.213 | -0.07752 | -0.08658 | -0.118 | -0.031 | -0.210 | -0.126 |
| **As** | -0.486 | -0.408 | 0.472868 | 0.324675 | 0.348 | 0.326 | 0.526 | 0.464 |
| **As+Si** | -0.284 | -0.199 | 0.197674 | 0.093074 | 0.496 | 0.568 | 0.228 | 0.267 |

**(B)**

| **Fold Change 7DAY** | | | | | | | | |
| --- | --- | --- | --- | --- | --- | --- | --- | --- |
|  | **SOD** | | **CAT** | | **GPX** | | **GST** | |
|  | **Shoot** | **Root** | **Shoot** | **Root** | **Shoot** | **Root** | **Shoot** | **Root** |
| **C** | 0 | 0 | 0 | 0 | 0 | 0 | 0 | 0 |
| **Si** | 0.184715 | 0.171942 | 0.044852 | 0.221637 | -0.03649 | -0.01543 | 0.069147 | -0.05046 |
| **As** | 0.709126 | 0.377033 | 0.39949 | 0.525405 | 0.349206 | 0.419511 | 0.238379 | 0.171742 |
| **As+Si** | 0.516216 | 0.230165 | 0.292469 | 0.4329 | 0.123903 | 0.248725 | 0.448487 | 0.306603 |
| **Fold Change 15DAY** | | | | | | | | |
|  | **SOD** | | **CAT** | | **GPX** | | **GST** | |
|  | **Shoot** | **Root** | **Shoot** | **Root** | **Shoot** | **Root** | **Shoot** | **Root** |
| **C** | 0 | 0 | 0 | 0 | 0 | 0 | 0 | 0 |
| **Si** | -0.187 | -0.07833 | -0.01444 | 0.239472 | -0.01081 | -0.00224 | 0.02507 | -0.04829 |
| **As** | 1.155358 | 0.54149 | 0.430098 | 0.444039 | 0.324717 | 0.537533 | 0.172046 | 0.112711 |
| **As+Si** | 0.863192 | 0.305361 | 0.63582 | 0.507438 | 0.423432 | 0.675569 | 0.429389 | 0.218577 |

(C)

|  | **Fold Change 7DAY** | | | | | |
| --- | --- | --- | --- | --- | --- | --- |
|  | **APX** | | **MDHAR** | | **GR** | |
|  | **Shoot** | **Root** | **Shoot** | **Root** | **Shoot** | **Root** |
| **C** | 0 | 0 | 0 | 0 | 0 | 0 |
| **Si** | -0.0263 | -0.01142 | 0.018227 | 0.45283 | 0.000542 | -0.03533 |
| **As** | 0.44017 | 0.561201 | 0.69725 | 0.45283 | 0.587708 | 0.505252 |
| **As+Si** | 0.51107 | 0.879423 | 0.493742 | 0.45283 | 0.448198 | 0.654463 |
|  | **Fold Change 15DAY** | | | | | |
|  | **APX** | | **MDHAR** | | **GR** | |
|  | **Shoot** | **Root** | **Shoot** | **Root** | **Shoot** | **Root** |
| **C** | 0 | 0 | 0 | 0 | 0 | 0 |
| **Si** | -0.00907 | -0.10921 | 0.004216 | 0.04491 | 0.009932 | -0.05564 |
| **As** | 0.534733 | 0.481123 | 0.752534 | 0.453608 | 0.934279 | 0.575708 |
| **As+Si** | 0.727511 | 0.703262 | 1.062367 | 0.563796 | 1.18672 | 0.702383 |

**Figure S1.** Relationship between percentage seed germination and time after sowing at different concentration of **(A)** As^(III)^ (50µM-Low; 150µM-Moderate; 300µM-High) **(B)** As^(III)^+Si (50µM+5mM-Low; 150µM+5mM-Moderate; 300µM+5mM-High).

**
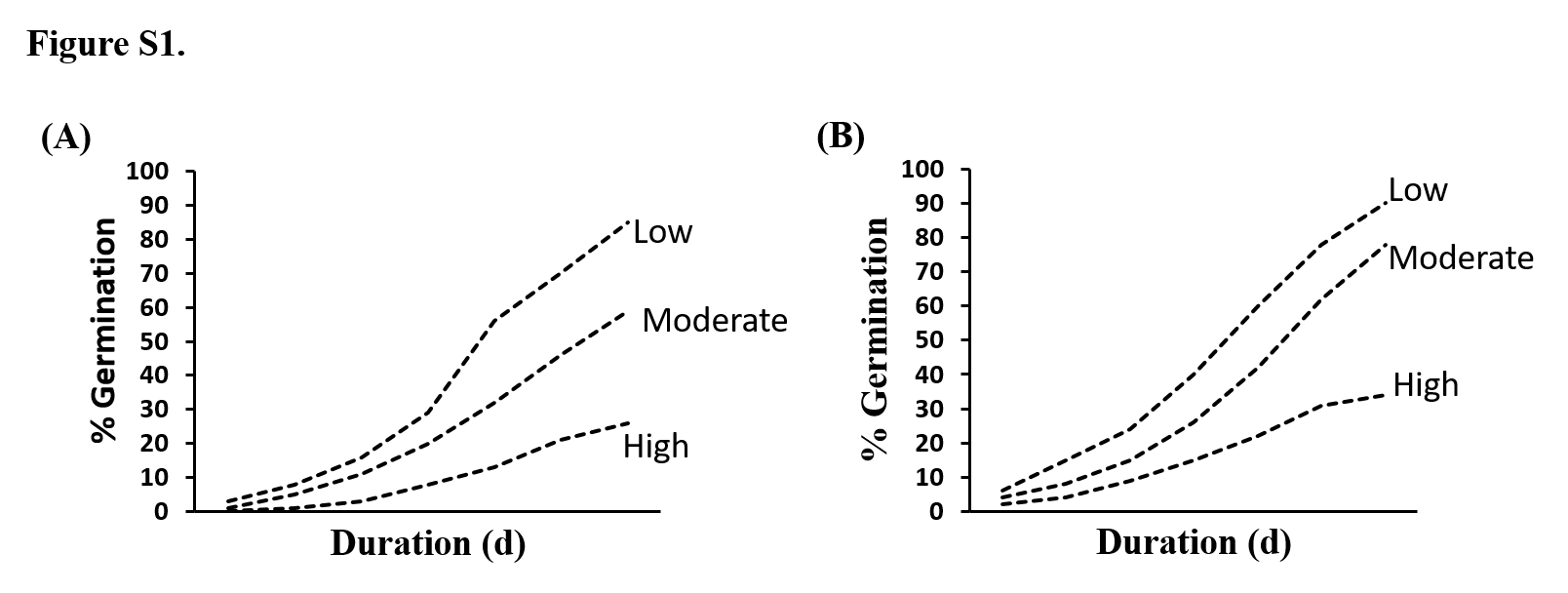
**

**Figure S2.** Phenotypic difference in shoot and root growth of rice var. IR-64 treated with As^(III)^ 150µM and Si 5mM alone and in combination after 7 and 15 d.


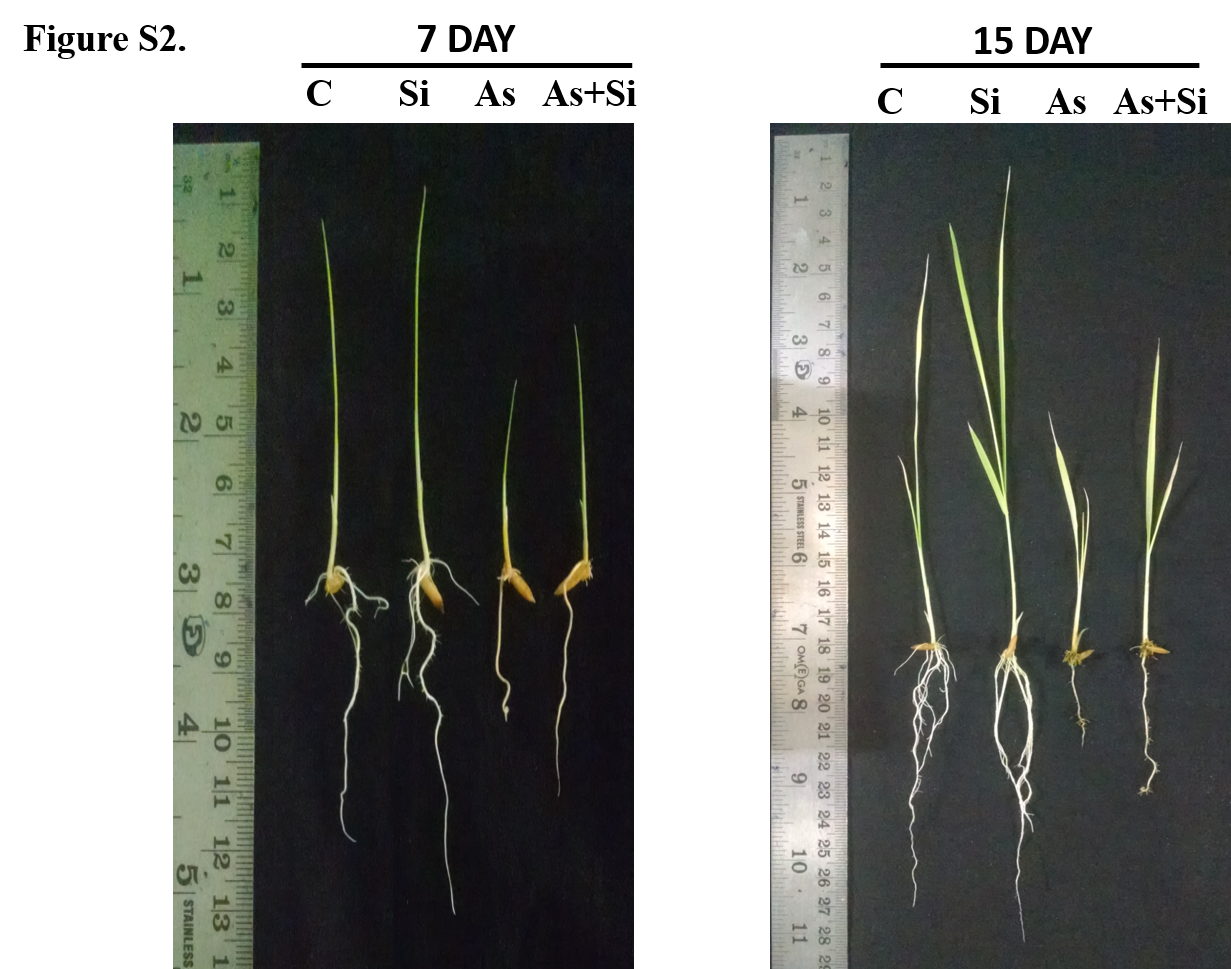

Supplement: Supplementary file 1 — Supplementary Information [file 41598_2018_28712_MOESM1_ESM.doc]
